# The Interplay between Environmental Filtering and Spatial Processes in Structuring Communities: The Case of Neotropical Snake Communities

**DOI:** 10.1371/journal.pone.0127959

**Published:** 2015-06-10

**Authors:** Hamanda Cavalheri, Camila Both, Marcio Martins

**Affiliations:** 1 Departamento de Ecologia, Instituto de Biociências, Universidade de São Paulo, 05508–090, São Paulo, SP, Brazil; 2 Programa de Pós-graduação em Biodiversidade animal, Universidade Federal de Santa Maria, 97105–900, Santa Maria, RS, Brazil; University of Brasilia, BRAZIL

## Abstract

Both habitat filters and spatial processes can influence community structure. Space alone affects species immigration from the regional species pool, whereas habitat filters affect species distribution and inter-specific interactions. This study aimed to understand how the interplay between environmental and geographical processes influenced the structure of Neotropical snake communities in different habitat types. We selected six studies that sampled snakes in forests, four conducted in savannas and two in grasslands (the latter two are grouped in a non-forest category). We used the net relatedness and nearest taxon indices to assess phylogenetic structure within forest and non-forest areas. We also used the phylogenetic fuzzy-weighting algorithm to characterize phylogenetic structure across communities and the relation of phylogenetic composition patterns to habitat type, structure, and latitude. Finally, we tested for morphological trait convergence and phylogenetic niche conservatism using four forest and four non-forest areas for which morphological data were available. Community phylogenetic composition changed across forest and non-forest areas suggesting that environmental filtering influences community structure. Species traits were affected by habitat type, indicating convergence at the metacommunity level. Tail length, robustness, and number of ventral scales maximized community convergence among forest and non-forest areas. The observed patterns suggested environmental filtering, indicating that less vertically structured habitats represent a strong filter. Despite the fact that phylogenetic structure was not detected individually for each community, we observed a trend towards communities composed by more closely related species in higher latitudes and more overdispersed compositions in lower latitudes. Such pattern suggests that the limited distribution of major snake lineages constrained species distributions. Structure indices for each community were also related to habitat type, showing that communities from non-forest areas tend to be more clustered. Our study showed that both environmental filtering and spatial gradients play important roles in shaping the composition of Neotropical snake communities.

## Introduction

One of the major challenges in ecology is to understand the processes that regulate species composition in different communities. Species diversity in communities results from two sets of forces: ecological processes, such as interactions between species and with abiotic factors, and biogeographical processes, such as species immigration, speciation, and extinction [1]. Competition and environmental filtering are two processes that are most commonly believed to be involved in the structuring of ecological communities [2–4]. Competition theory predicts that, in order to avoid competitive exclusion, species with similar niches can only coexist when there is spatial or temporal niche partitioning or character displacement [5–8]. Hence, communities structured by competition tend to be characterized by overdispersed traits. On the other hand, environmental constraints filter species that have the ability to survive in a given place, thus favoring species with similar ecological requirements [9,10]. In this case, communities display a clustered trait pattern. These two contrasting pressures may operate simultaneously and communities may be structured by clustered and overdispersed trait patterns concomitantly [11]. Furthermore, spatial and temporal scales must be considered when interpreting the patterns found. For instance, an overdispersed trait pattern may arise due to environmental heterogeneity at small scales or a clustered pattern may be mediated by temporal fluctuations [12,13]. An alternative theory suggests that neither one of these two ecological processes can influence community structure, and that species abundance is the result of ecological drift [14], or that biotic interactions and environmental filtering might balance each other out, resulting in a random or neutral pattern in community structure [15].

Regardless of which ecological process drives community assembly, the evolutionary history of lineages is expected to play an important role in structuring communities and has received increasing attention from ecologists. Evolutionary history is believed to largely influence phenotypic similarity across lineages [16,17]. Phenotypic traits are the means by which species interact with their environment, acting on both species interactions and habitat filtering. In addition, the evolutionary history of lineages is expected to limit the geographical distribution of species [18]. For instance, if clades have dispersal restrictions due to factors that vary with latitude (e.g. climate), a filter (e.g. habitat type) can act only on species that were able to disperse to that community [1]. Given the importance of the evolution of clades in shaping communities, the challenge is to understand how environmental, historical, and spatial factors interplay in structuring communities.

For vertebrates, major morphological changes are a result of ecological aspects, mainly habitat use [19–23]. Snakes are known to have body shape and other morphological characters highly related to habitat use, which is evident despite their limblessness [23,24]. Body size, stoutness, body elongation, and presence of cephalic scales are good examples of traits that could be associated with habitat use by snakes [22–25]. These features make them useful model organisms to understand the influence of vegetation type on community structure.

Moreover, it has been suggested that snake species occupying the same habitat might be under the same selective pressures, which tends to generate convergent morphologies, independent of lineage [26,27]. Environmental filtering is claimed to be a key process driving community assembly when distantly related species experiencing similar environmental conditions present similar trait values [17,28–30]. However, whether phylogenetic structure and trait convergence in snake communities are related to habitat use (i.e. environmental filtering) or primarily reflect evolutionary history remains to be investigated.

Within this context, our aim in this study is to understand the influence of habitat type and latitudinal spatial gradient on the structure of Neotropical snake communities occurring in forest and non-forest habitats. We addressed the following questions: 1. Are communities phylogenetically structured by environmental filtering and/or latitudinal gradients? 2. Are habitat types filtering convergent morphologies? 3. Should this be the case, is morphological convergence related to habitat filtering and independent of species ancestral morphology? We expected habitat type to have a strong influence on the phylogenetic structure of snake communities. Accordingly, we expected communities from non-forest areas to be phylogenetically clustered, since they occur in a less vertically structured habitat. In contrast, communities from forest areas should exhibit overdispersed phylogenetic compositions because they occur in a more vertically complex and heterogeneous habitat. Furthermore, snakes from forests and non-forest areas should possess different trait values, indicating that vegetation types filter similar morphologies. We also expected closely related species to display more similar trait values than distantly related species.

## Materials and Methods

### Species Occurrence Data

**Fig 1:**
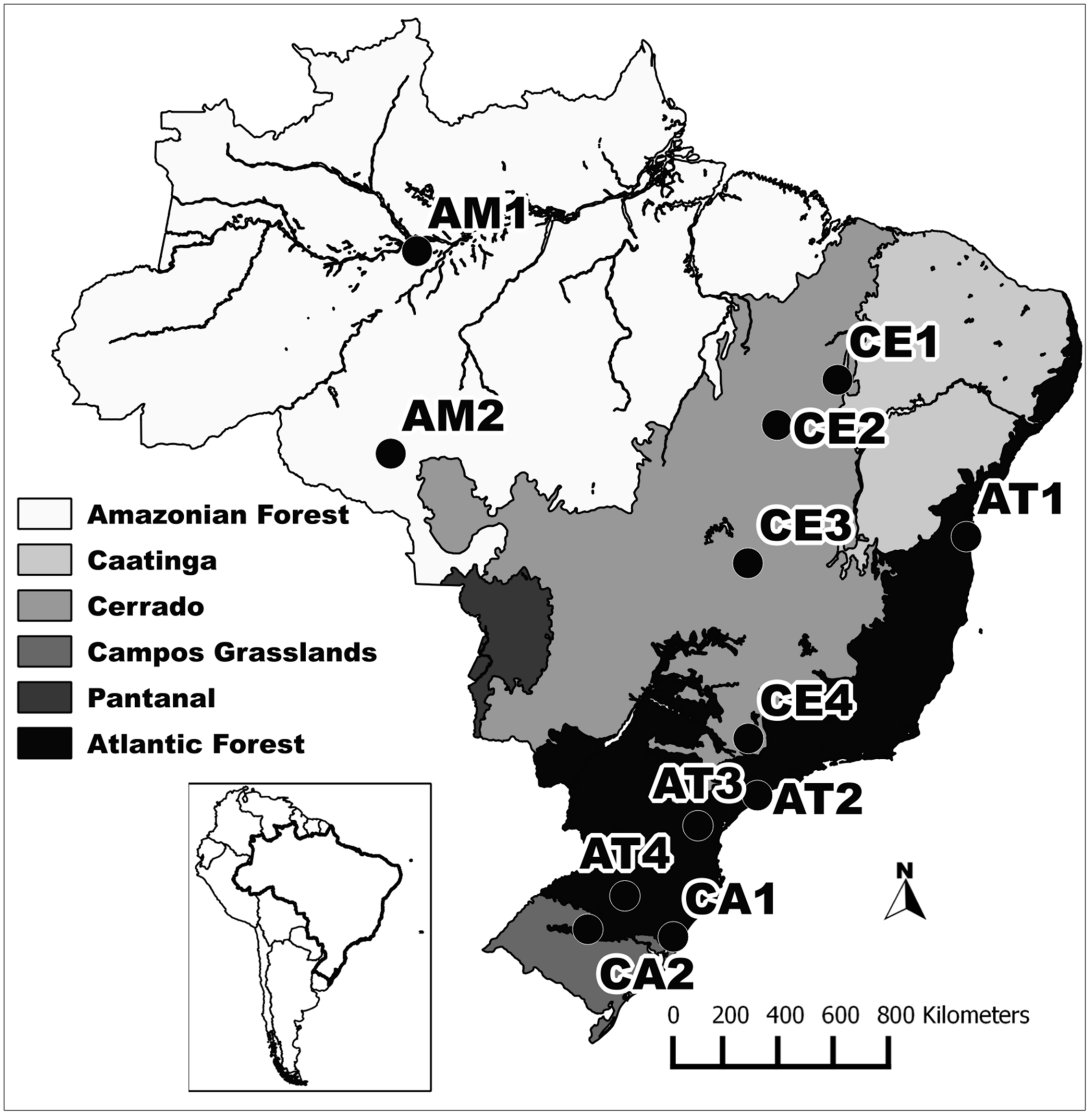
Map of Brazil indicating snake communities studied. Background colors represent the biomes to which communities belong.

We compiled a database of species composition for snake communities in Brazil using lists from published references in peer-reviewed journals and unpublished data provided by other researchers (S1 Table). In both cases, we used exclusively studies in which local species richness was well sampled (i.e., species accumulation curves reached, or were close to reaching, saturation). Data comprised communities from four major vegetation types, including forests (two communities from Amazon Forest and four from Atlantic Forest) and more open, less vertically structured vegetation types (herein referred to as non-forest areas) such as savannas and grasslands (four communities from Cerrado in central Brazil and two from Campos in the south; Table 1; Fig 1). We grouped these non-forest areas based on the similarity in composition of their snake faunas.

**Table 1:** Geographical coordinates and source of data for species composition of each snake community considered in this study.

Community	Geographical Coordinates	Reference
Amazonian 1	3°6’S; 60°1’W	[53]
Amazonian 2	11°31’S; 61°1’W	[54]
Atlantic Forest 1	14°47’S; 39°2’W	[59]
Atlantic Forest 2	24°32’S; 47°15’W	[51]
Atlantic Forest 3	25°47’S; 49°41’W	[60]
Atlantic Forest 4	28°14’S; 52°19’W	[61]
Cerrado 1	08°50’S; 44°10’W	[62]
Cerrado 2	10°22’S; 46°40’W	[63]
Cerrado 3	15°48’S; 47°51’W	[64]
Cerrado 4	22°15’S; 47°49’W	[49]
Campos 1	29°26’S; 50°35”W	[65]
Campos 2	29°41’S; 53°48’W	[66]

We included all species found in all areas sampled but considered only presence and not abundance. Some studies included both forest and non-forest areas, and in such cases we included those species observed in only one of the areas, according to the major vegetation type we are considering (e.g. if the focus of study was Atlantic Forests, we did not include snakes found in open areas within Atlantic Forests). However, a few species that occur mainly in the gallery forests in Cerrado areas (e.g., *Bothrops moojeni*) were treated as Cerrado species, since they may occasionally be found in Cerrado vegetation. Moreover, given that we seek processes that relate species composition and vegetation type, we chose not to include aquatic species.

### Species Morphological Traits

We only used adult female specimens from localities within a latitudinal range of 5° north and south of the location where the community was studied (S1 Table). We used exclusively females to avoid bias introduced by tail measurements of males, which tend to have longer tails because of the presence of hemipenes. For each individual, we measured snout—vent length (SVL), tail length (TL), circumference around midbody (CAM, which indicates stoutness after controlling for body size), head length (HL, tip of snout to posterior edge of mandible), head width (HW, at posterior edge of mandible), head height (HH, at its highest point), ventral scale count (VS, as a surrogate for the number of body vertebrae), and subcaudal scale count (SS, as a surrogate for the number of tail vertebrae). We measured these traits in specimens of nine scientific collections in Brazil under the care of the following curators: F. L. Franco (Instituto Butantã—IB), H. Zaher (Museu de Zoologia da Universidade de São Paulo—MZUSP), F. Toledo and P. R. Manzani (Museu de Zoologia da Universidade Estadual de Campinas—ZUEC), T. Grant and G. M. F. Pontes (Museu de Ciência e Tecnologia da Pontifícia Universidade Católica do Rio Grande do Sul—MCT), S. Cechin (Coleção Herpetológica da Universidade Federal de Santa Maria—ZUFSM), G. Colli (Coleção Herpetológica da Universidade de Brasília—CHUNB), A. L. Prudente (Museu Paraense Emílio Goeldi—MPEG), A. J. S. Argôlo (Museu de Zoologia da Universidade Estadual de Santa Cruz—MZUESC, and Coleção Zoológica Gregório Bondar—CZGB) (S1 File). We calculated average values for each trait and each species represented by two or more individuals (S2 Table).

### Phylogenetic Relationships

**Fig 2:**
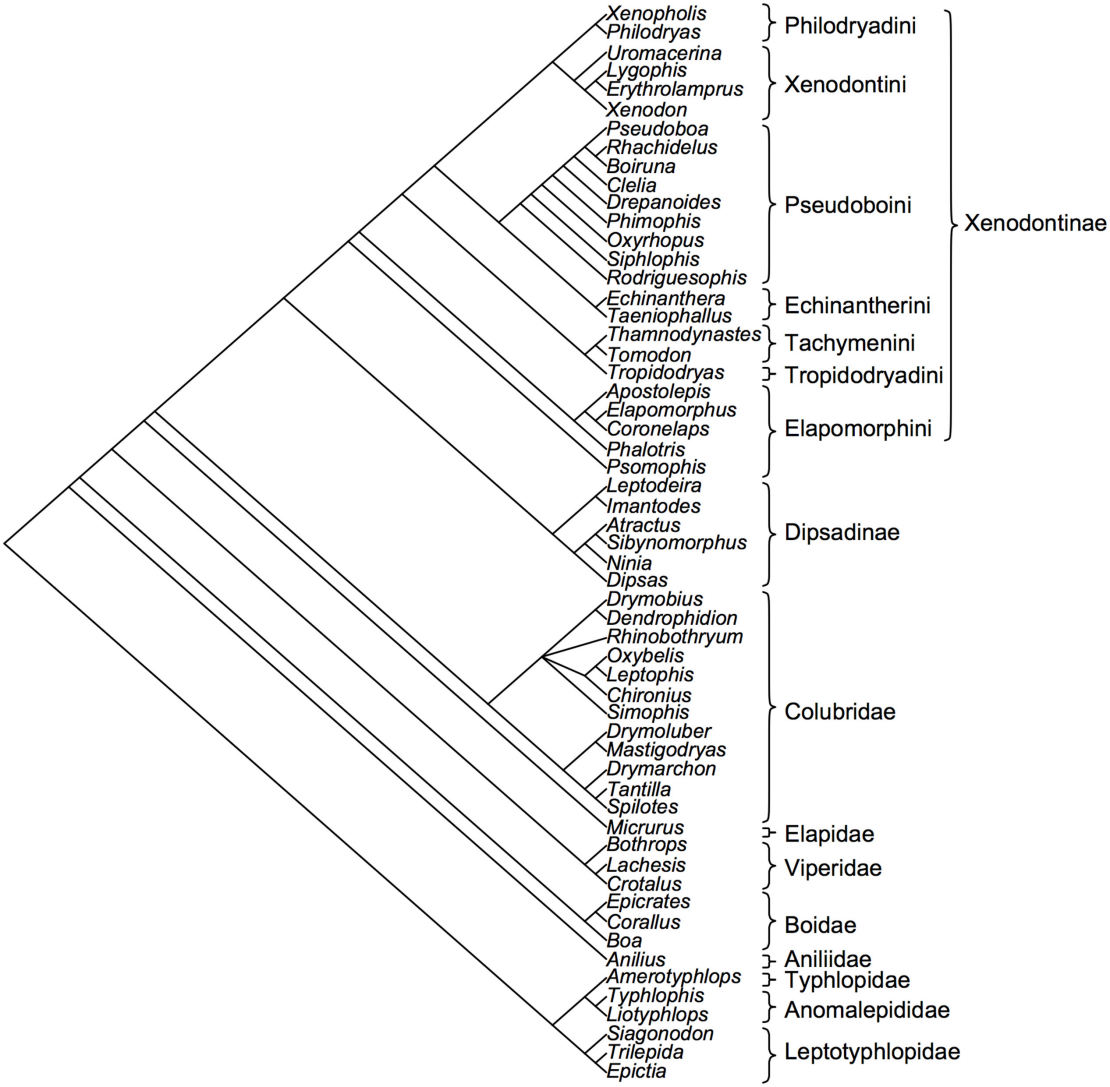
Phylogenetic tree for snake genera occurring in the eight communities studied. Phylogeny derived from multiple sources (see S2 File). Branch lengths are only illustrative. Major lineages follow [58].

We constructed a topological phylogenetic tree (Fig 2, S1 Fig) including all species from each community, and individual phylogenies for each community, including species of their respective regional species pool (i.e. the species that could potentially compose the communities; see below), based on published phylogenies and personal communications from specialists (S2 File), using the software Mesquite 2.75 [31]. Because branch lengths were not available, analyses were performed by node counting.

### Data Analyses

First, we analyzed phylogenetic structure within each community using the net relatedness (NRI) and nearest taxon (NTI) indices [2]. NRI is derived from the mean pairwise distance between all species in a community, and NTI is derived from the mean distance separating each species in the community from its closest relative. Therefore, NRI is more sensitive to tree-wide patterns, while NTI is more sensitive to patterns closer to the tips of the phylogeny. To obtain the significance of observed results, we generated null communities by randomizing (9,999 randomizations) the tip labels of the phylogeny, holding species richness constant. Positive NRI and NTI values indicate phylogenetic clustering, whereas negative values indicate phylogenetic overdispersion. These two standardized indices follow a z distribution; thus, if either index has values greater than 1.96, the community has a statistically significant clustered pattern. In contrast, if values are less than -1.96, the community has a statistically significant overdispersed pattern. Communities with non-significant NRI and NTI values are assumed to be phylogenetically random. We used subtrees containing each community’s regional species pool (i.e. species that could potentially compose the community) for these analyses. To determine the regional species pool for each community, we first created a buffer of 500 km around the location where the community was studied. Owing to differences in vegetation types within a biome (like the Amazon forest, for instance, where patches of open formations may be found) the species pool range was superimposed on a map of terrestrial ecoregions of the world [32]. Ecoregions in which each Brazilian species occurred were determined by experienced researchers; for Bolivian and Argentinian species, we gathered information from the literature (for Argentina: [33], for Bolivia: [34,35]). Next, we used linear partial regression [36] to compare NRI and NTI values from different communities (forest and non-forest areas) and latitudes. Accordingly, we evaluated the independent contributions of habitat types and space, as well as the spatial structuring of habitats.

Second, we assessed phylogenetic structure among communities (i.e. at the metacommunity level) using phylogenetic fuzzy-weighting [37]. To obtain a phylogeny-weighted species composition matrix (matrix **P**), we weighted species composition according to phylogenetic similarity through a fuzzy set algorithm [37]. The resultant matrix **P** contains all species versus locality associations as in the original species composition matrix, but taking into account species shared phylogenetic similarity. We then submitted matrix **P** to principal coordinate analysis (PCoA) [36] to obtain the principal components of phylogenetic structure (PCPS), which represent orthogonal vectors describing gradients for the metacommunity and indicate which clades are most strongly associated with it [38]. The ordination was carried out using Bray-Curtis as a dissimilarity measure. The ordination resulted in seven orthogonal vectors describing community gradients. Some of these gradients are expected to relate to unmeasured factors and could introduce noise into the analysis [39]. To select only gradients related to vegetation types and space variables, we adopted the criteria proposed by Duarte et al. [39], choosing the number of PCPS that minimizes the residual sum of squares when relating phylogenetic composition to explanatory variables. We performed multiple db-RDA ordination relating gradients to predictors, successively increasing the PCPS number, and observed F-values. The first two PCPS, which explained 74% of the variation, held the greatest F-value (i.e. lower residual variation) when related to habitat types and latitude. We assessed the independent and shared contributions of habitat types and latitude to phylogenetic composition structure (PCPS1 and PCPS2) using partial linear regression.

Finally, we tested if snake communities show morphological trait convergence patterns related to habitat filtering, and if such convergence is independent of phylogenetic relationships. For these analyses we used only eight communities for which we could collect morphological data: Campos and Amazon communities and Cerrado 2, Cerrado 3, Atlantic 1 and Atlantic 2 (see Fig 1 and Table 1). We tested trait convergence using the correlation between dissimilarity matrices of mean trait values for each community (matrix **T**) and habitat types [37]. If communities show trait convergence linked with habitat type, we expect this correlation to be strong [37]. A matrix **T** describing trait average values for communities was obtained through multiplication of two matrices: a matrix of traits by species, and the standardized matrix of species composition by community totals. Since trait structure of communities could reflect both convergence and divergence patterns, we only used traits that maximize convergence. Traits maximizing convergence were selected by iterative searching using the *optimal* function in the *Syncsa* R package [11]. The significance of correlations between dissimilarity matrices was tested against null models using 9,999 permutations.

Snake communities presented morphological convergence owing to habitat type (see Results). Accordingly, we used a d-separation model proposed by Pillar and Duarte [37] to test if trait convergence reflects phylogenetic niche conservatism or is independent of phylogeny. Considering that i) similar habitats will select species with similar phenotypic traits (*ρ*(**PE**)), and ii) species traits are expected to be phylogenetically conserved (*ρ* (**PT**)), iii) the relationship between morphological traits and habitats is expected to be mediated by phylogeny (E→P→T). If morphological convergence owes to phylogenetic niche conservatism, the relationship between community mean trait values and habitat types must be null after controlling for the phylogenetic structure of the community *ρ*(**TE.P**) [34]. After controlling for phylogenetic structure, the remaining trait variation is causally independent of phylogeny. The significance of correlations between dissimilarity matrices was tested against null models using 999 permutations.

All analyses described above were performed in R 2.15 [40]. Specifically, NRI and NTI were calculated using the *picante* package [41]. We used the *Syncsa* package to scale-up phylogenetic and trait data into the species composition matrix and to test for phylogenetic signal [42]. Partial regressions were performed using the *varpart* function of the *vegan* package [43].

## Results

Our database contained 182 snake species (S1 Table). Total local richness for forests (126 species) was almost 1.5 times greater than for non-forest areas (85 species), with some species occurring in both areas. Specifically, richness for Amazonian communities was 61 (Amazonian 1) and 45 (Amazonian 2) species, whereas Atlantic communities possessed 55 (Atlantic 1), 28 (Atlantic 2), 20 (Atlantic 3) and 13 (Atlantic 4) species (Table 2). For non-forest areas, richness for Cerrado communities was 24 (Cerrado 1), 27 (Cerrado 2), 42 (Cerrado 3) and 29 (Cerrado 4) species, whereas Campos communities possessed 9 (Campos 1) and 18 (Campos 2) species (Table 2).

**Table 2:** Richness and phylogenetic community structure indices for each community studied.

Community	Richness	NRI	NTI
Amazonian 1	61	-1.821	0.607
Amazonian 2	45	-1.431	1.413
Atlantic 1	55	-0.054	1.709
Atlantic 2	28	0.359	1.030
Atlantic 3	20	1.220	-0.592
Atlantic 4	13	0.931	0.932
Cerrado 1	24	-0.488	-0.130
Cerrado 2	27	-0.794	0.275
Cerrado 3	42	0.941	0.253
Cerrado 4	29	1.149	-1.173
Campos 1	9	**2.743**	1.282
Campos 2	18	1.718	0.654

NRI and NTI > 1.96 indicate phylogenetic clustering. NRI and NTI < -1.96 indicate phylogenetic overdispersion. Values in bold are statistically significant results.

**Fig 3:**
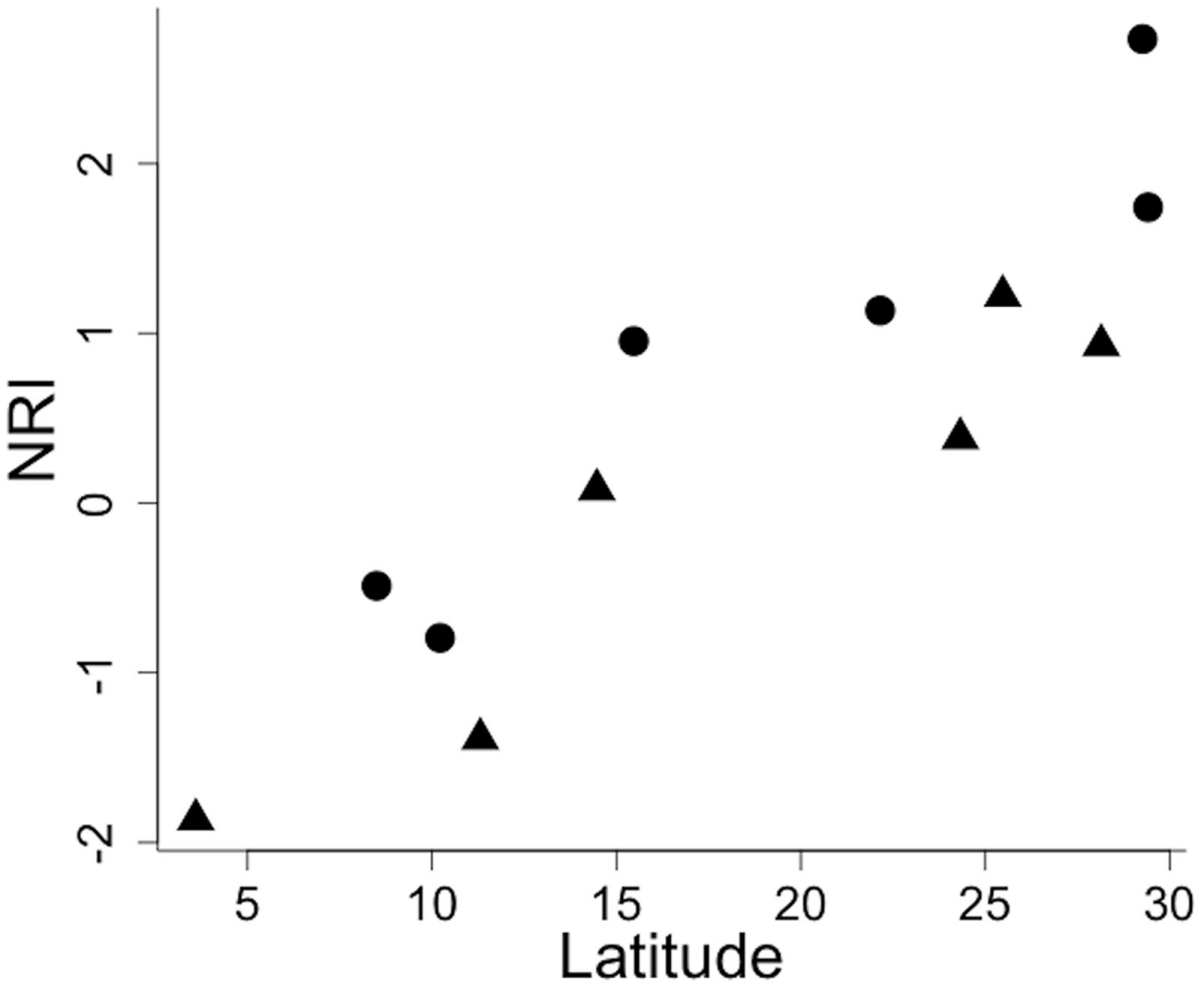
Plot of NRI in relation to latitude. Triangles represent forest areas and circles represent non-forest areas.

We found significant phylogenetic clustering in only one community, Campos 2 (Table 2). Results from all other communities were non-significant, indicating phylogenetic randomness. Habitat type and latitude accounted for 87% of the variation in NRI values (R^2^ = 0.897, F_2,9_ = 37.27, p = 0.005). Latitude independently explained 80% of NRI variation. Snake communities of higher latitudes possessed greater NRI values, indicating a trend of clustered patterns, whereas communities of lower latitudes tended to show overdispersed patterns (Fig 3). Habitat accounted for 9% of NRI variation. Communities from non-forest areas tended to be more clustered. None of the communities studied had significant NTI values (Table 2). Furthermore, the linear regression showed that neither latitude nor vegetation types explained NTI values of snake communities (R^2^ = 0.168, F_2,9_ = 0.915, p = 0.435).

**Fig 4:**
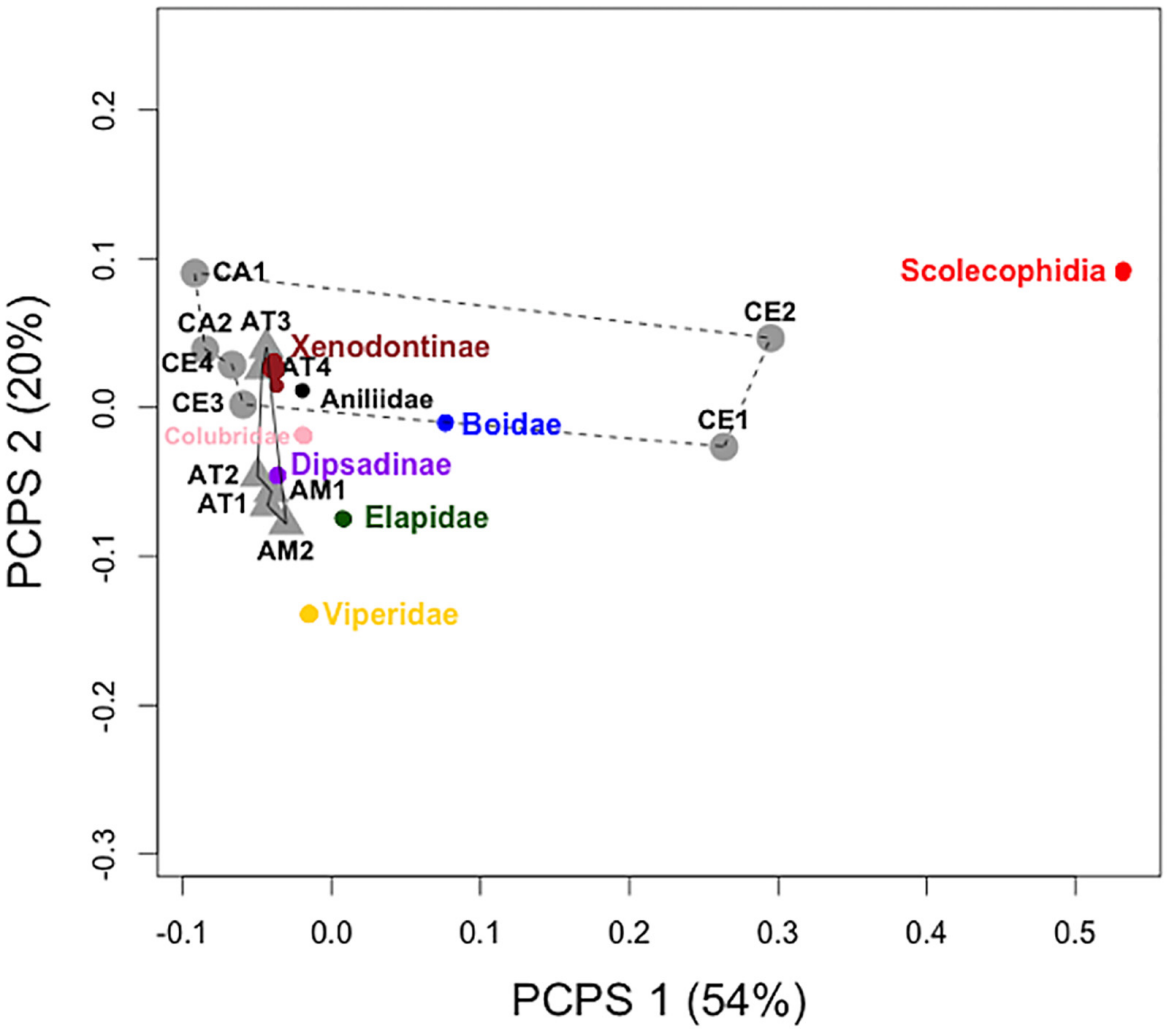
Ordination plot showing principal coordinates of phylogenetic structure (PCPS) of snake communities occurring in forest (gray triangles) and non-forest areas (gray circles). Dots represent species scores, and dot colors match lineage colors name. Lines connect communities of the same type of vegetation, with solid lines for forest and dashed lines for non-forest vegetation. Scolecophidia is composed by families Leptotyphlopidae, Anomalepididae, and Typhlopidae.

The ordination of the matrix **P** resulted in seven axes, with the first two axes describing 74% of the variability (Fig 4). PCPS1, describing 54% of variation, showed no significant relationship with habitat type and latitude (R^2^ = 0.004, F_2,9_ = 0.018, p = 0.98). The ordination plot showed that the phylogenetic compositions of CE1 and CE2 are distinct from all the remaining communities along PCPS1 (Fig 4). It is also possible to observe that Scolecophidia, the most basal clade, was separated from all remaining lineages. The second axis describing phylogenetic structure, PCPS2, was explained only by habitat type (R^2^ = 0.51, F_1,10_ = 12.3, p = 0.006; Fig 4). Species of the families Viperidae and Elapidae and from the subfamily Dipsadinae were closely associated with forest communities. Species of the subfamily Xenodontinae appear more associated to communities of lower latitudes, being mainly close to Atlantic communities (AT3 and AT4).

**Fig 5:**
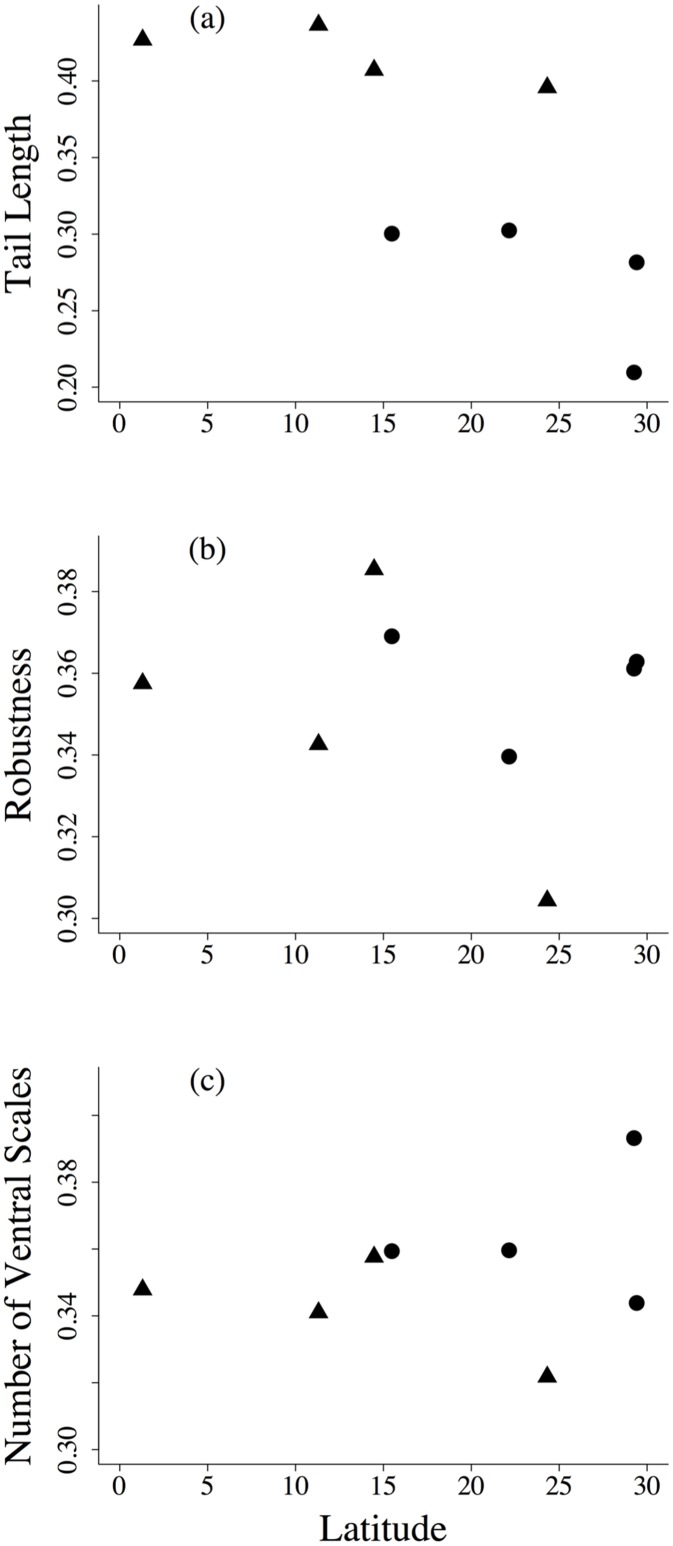
Plot of tail length (a), robustness (b), and number of ventral scales (c) in relation to latitude. Triangles represent forest areas and circles represent non-forest areas.

Community composition weighted by mean trait values was significantly correlated with habitat type (*ρ*(TE) = 0.572, p = 0.049), indicating morphological convergence at the metacommunity level. This result was obtained from the subset of communities with phenotypic data available (8 out of 12 communities). Among the eight morphological traits measured, tail length, robustness, and number of ventral scales maximized community convergence related to habitat type (Fig 5). Communities from forests comprised species with longer SVL, TL, and HL. They were also characterized by species with fewer ventral and more subcaudal scales than species from non-forest areas. Furthermore, species from non-forest areas were characterized by larger body diameters. Community trait structure was correlated to phylogenetic structure (*ρ*(PT) = 0.733, p = 0.1.) In addition, D-separation analysis indicated that community trait convergence across habitat depends on phylogenetic composition (*ρ*(TE.P) = 0.303, p = 0.128; alternative hypothesis), supporting the phylogenetic niche conservatism hypothesis.

## Discussion

These results corroborate our initial hypotheses, showing that habitat filtering plays an important role in the phylogenetic structure of Neotropical snake metacommunities. Community trait patterns were related to habitat type, indicating that compositions inhabiting the same vegetation types possessed convergent morphological traits. Such convergent phenotypic patterns are mediated by phylogenetic composition of communities, suggesting that habitat gradient favors phylogenetically conserved traits. Overall, we could not support phylogenetically clustered or overdispersed patterns within most communities. However, spatial gradients, herein measured by latitude and habitat type explained the variation of NRI values, suggesting phylogenetic structure at the metacommunity scale. In sum, our results suggest an interplay between environmental filters and spatial gradients has shaped phylogenetic and functional compositions of Neotropical snake communities. These results agree with other recent studies that found that the interplay between biogeographical and environmental factors has shaped community structure (e.g. [44–46]).

Community analyses revealed that most communities could be considered as randomly structured phylogenetically, except for one case. However, when we compared their individual structure descriptors, we observed that communities from lower latitudes tend to exhibit a pattern of phylogenetic overdispersion, whereas those from higher latitudes tend to be phylogenetically clustered. These results might be related to the number of species available to become members of a given community (i.e. the size of the regional species pool). Indeed, the three main South American snake lineages (Colubridae, Dipsadinae, and Xenodontinae) have distinct geographic distribution patterns [22]. Clade distribution patterns can relate to differences in center of origin, ancestral ecological niche, dispersal limitation, and time for speciation [47]. The diversity of dipsadines is higher in Central America, that of xenodontines is higher in the southern part of South America, and colubrids are most diverse in North America [22]. As a result, South American snake communities from different latitudes tend to present different ratios of these lineages [22], with the diversity of colubrids and dipsadines decreasing southward, and that of xenodontines increasing northward, e.g. [48,49]). Hence, there is an overlap in the distribution of lineages in northern South America, which could explain the trend for phylogenetic overdispersion in communities at lower latitudes and phylogenetic clustering in communities at higher latitudes. Despite the fact that we could not relate spatial gradients with phylogenetic structure at the metacommunity scale, the ordination of phylogenetic composition showed dipsadines closer to Amazonian and Atlantic Forest communities and xenodondines closer to Campos and southern Atlantic Forest communities.

Additionally, the separation between Atlantic Forest communities in two groups might be related to the interruption of the Atlantic corridor in coastal Brazil during the Pleistocene [50] that prevented species from dispersing between the northern and southern portions of the Atlantic Forest. As a result, communities from southern Atlantic forest are more phylogenetically clustered than the northern one, for their composition is based primarily on one single lineage, xenodontines. Although xenodontines are mainly terrestrial and fossorial, there are arboreal and semi-arboreal species in the group, specialized in using arboreal substrates, in the southern Atlantic forest community [49,51,52]. Despite this diversity in habitat use, all species belong to the same lineage, resulting in a clustered phylogenetic structure in the community from the southern Atlantic Forest.

Most important, our results showed that communities present a similarity in phylogenetic composition related to the type of vegetation, which suggests that environmental filtering have shaped patterns of snake communities of non-forest habitats [4]. This might occur because arboreal and semi-arboreal species are less prone to occupying less vertically structured vegetation types due to the lower abundance of adequate substrate. Additionally, forested areas contained many arboreal and semi-arboreal species (e.g. *Dipsas*, *Sibynomorphus* and *Leptodeira* [51–54]), lineages that were absent from non-forest areas. That is, habitat structure seems to act as a lineage filter and play an important role in assembling snake community composition in the Neotropics [38,55,56]. Accordingly, we found a trend towards more clustered assembly patterns in non-forest areas than in forests when we analyzed the individual structure for each community.

Phylogenetic signal at the metacommunity level was also detected, which means that communities inhabiting the same type of vegetation have similar average trait values [37]. However, trait convergence is correlated to phylogenetic composition as well, which means that phylogenetically close species share more similar trait values than those that are phylogenetically distant. In this study, communities with the same type of vegetation have the same phylogenetic structure, but this does not mean that they are composed of the same species. In fact, this owes to three individual snake lineages occurring in the Neotropics and their distinct influence over the composition of communities (see above); therefore, we can infer that traits associated with habitat use are convergent across lineages. Previous studies have shown that snake morphology frequently reflects convergence across lineages as a result of distantly related species experiencing similar ecological conditions, which is corroborated by our finding that communities from forests are composed of species with longer SVL, TL, and HL than those from non-forest areas (e.g. [23,25]). This correlates with morphological syndromes related to habitat use in snakes. Arboreal snakes tend to be slender and have a laterally flattened body, longer tail, and more subcaudal scales, which provides greater mobility in a discontinuous substrate [23,24,57]. In contrast, terrestrial snakes tend to exhibit a generalist morphology, being stouter than arboreal species; indeed, we found that species are stouter in non forest areas than in forests. Therefore, we suggest that trait convergence across habitat types is consistent with environmental filtering. For instance, dipsadines are likely to occur in lower latitude communities and most of them use the arboreal substrate. This possibly limited their occupation of non-forest communities and, due to dispersal ability or other environmental filters, they may not be able to reach forest areas in higher latitudes. In southern communities, most arboreal species belong to Xenodontinae. This case exemplifies trait convergence across communities in our study.

In conclusion, habitat use affects the structure of Neotropical snake communities. Communities from forest and non-forest areas showed distinct phylogenetic-morphological compositions, and they also differed regarding intra-community assembly pattern. Thus, habitat structure seems to select which species occur in a given location depending on their traits and history. Latitude was not related to phylogenetic composition, however it explained variation in NRI values. We suspect that biogeographical descriptors, like the center of origin of the clades, could better explain phylogenetic composition at the metacommunity scale. Future studies should seek other trait variables (e.g. diet) and explore which biogeographical processes could act in association with environmental filtering on snake community structure in the Neotropics.

## Supporting Information

S1 Fig Topology. (PDF)

S1 File Specimens examined. (PDF)

S2 File Construction of snake topology. (PDF)

S1 Table Community composition. (PDF)

S2 Table Species traits. (PDF)
