# Supplementary material for: The Interplay between Environmental Filtering and Spatial Processes in Structuring Communities: The Case of Neotropical Snake Communities
Source: PLoS One. 2015 Jun 10;10(6):e0127959. doi: 10.1371/journal.pone.0127959 (PMC4465701; doi:10.1371/journal.pone.0127959)
Supplement: S1 Fig — (PDF) [file pone.0127959.s001.pdf]

S4 Fig. Topology

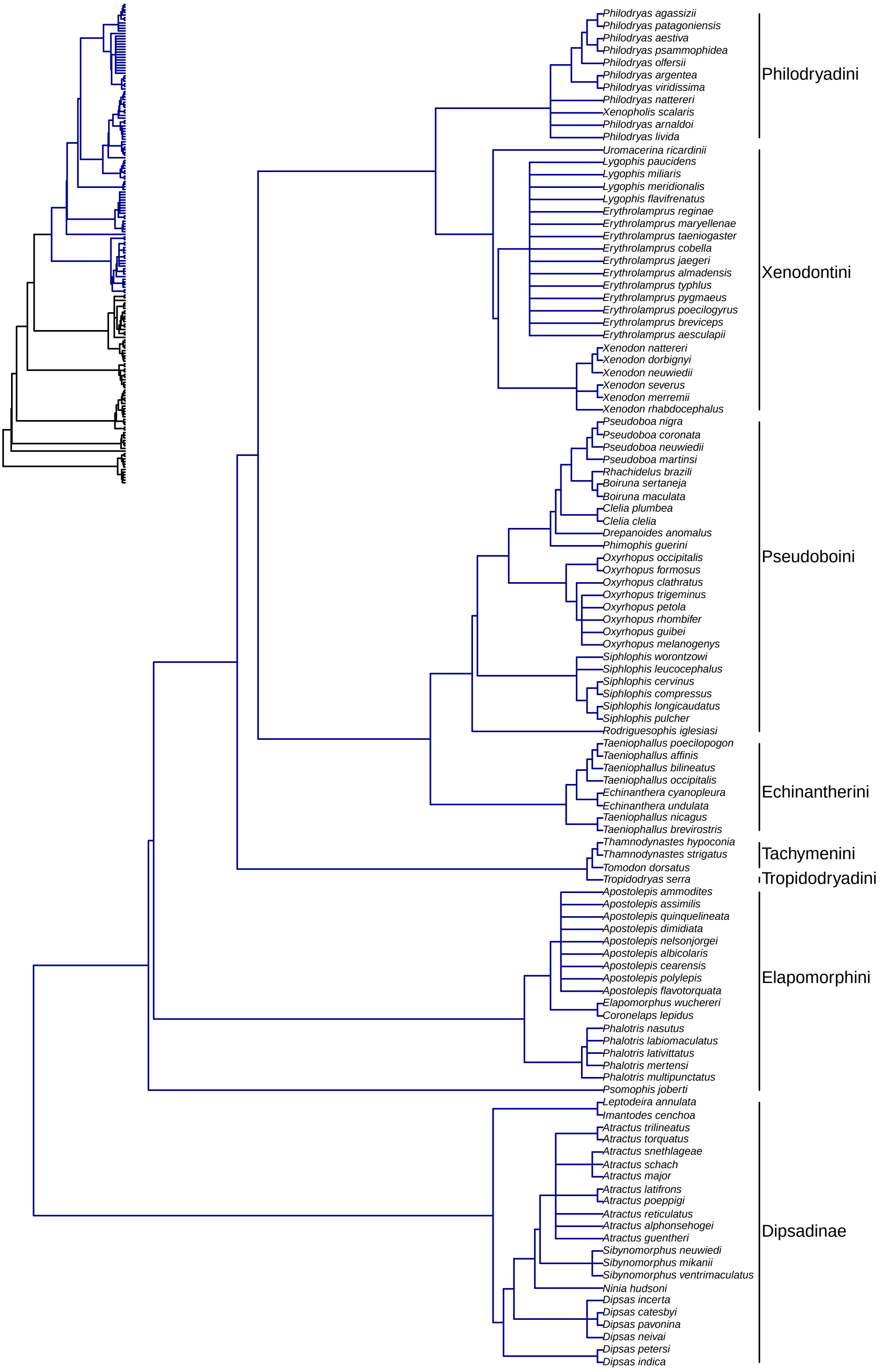

Topology of 182 snake species. The topology was compiled from multiple sources (see S5 File). Colors of clades indicate their position in the overall phylogeny, shown at left. Blue clade is Dipsadidae. Lines indicate family, subfamily or tribe. Branch lengths are only illustrative.

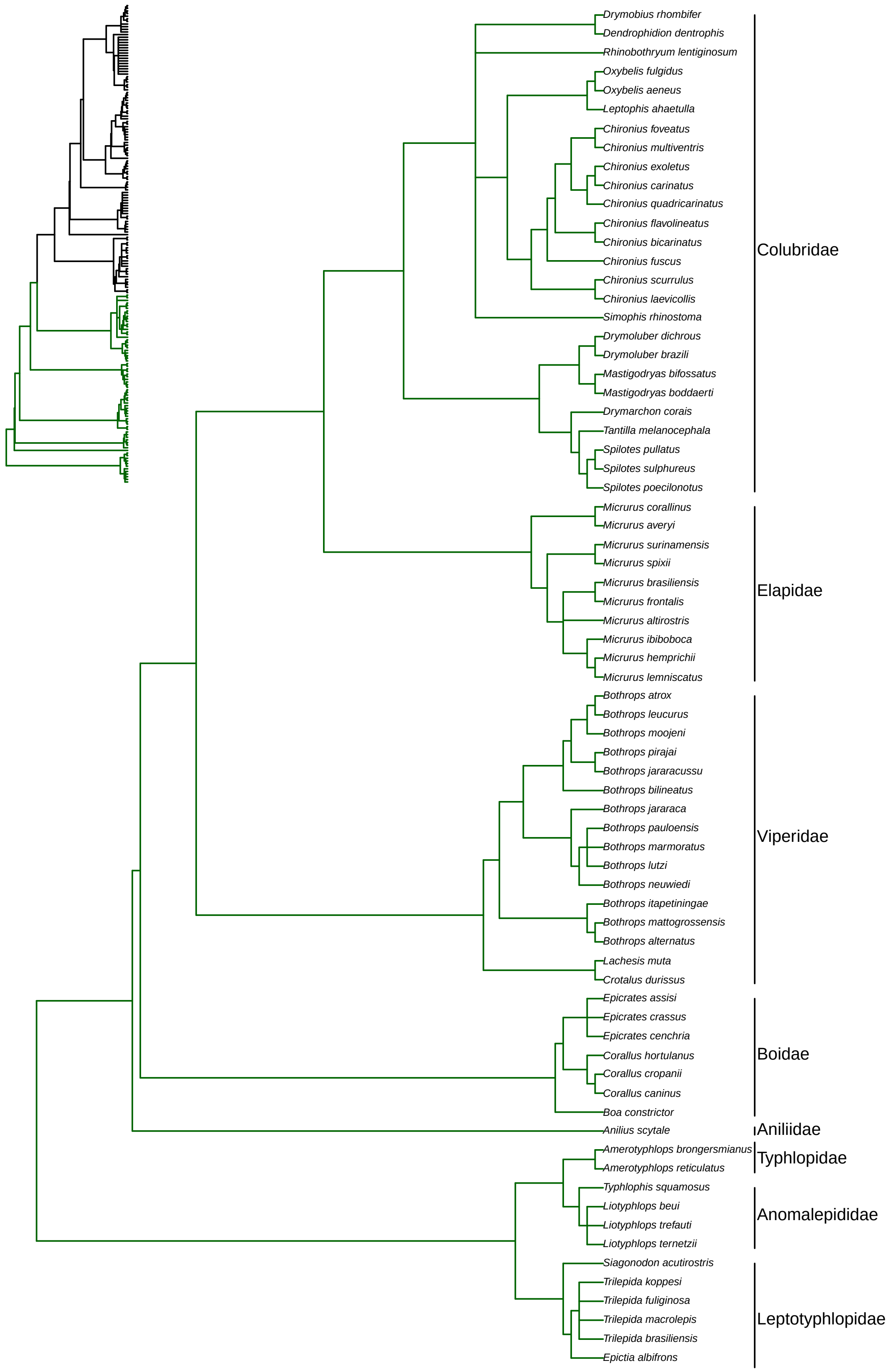

Drymobius rhombifer
Dendrophidion dentrophis
Rhinobothryum lentiginosum
Oxybelis fulgidus
Oxybelis aeneus
Leptophis ahaetulla
Chironius foveatus
Chironius multiventris
Chironius exoletus
Chironius carinatus
Chironius quadricarinatus
Chironius flavolineatus
Chironius bicarinatus
Chironius fuscus
Chironius scurrulus
Chironius laevicollis
Simophis rhinostoma
Drymoluber dichrous
Drymoluber brazili
Mastigodryas bifossatus
Mastigodryas boddaerti
Drymarchon corais
Tantilla melanocephala
Spilotes pullatus
Spilotes sulphureus
Spilotes poecilonotus
Colubridae
Micrurus corallinus
Micrurus averyi
Micrurus surinamensis
Micrurus spixii
Micrurus brasiliensis
Micrurus frontalis
Micrurus altirostris
Micrurus ibiboboca
Micrurus hemprichii
Micrurus lemniscatus
Elapidae
Bothrops atrox
Bothrops leucurus
Bothrops moojeni
Bothrops pirajai
Bothrops jararacussu
Bothrops bilineatus
Bothrops jararaca
Bothrops pauloensis
Bothrops marmoratus
Bothrops lutzi
Bothrops neuwiedi
Bothrops itapetiningae
Bothrops mattogrossensis
Bothrops alternatus
Lachesis muta
Crotalus durissus
Viperidae
Epicrates assisi
Epicrates crassus
Epicrates cenchria
Corallus hortulanus
Corallus cropanii
Corallus caninus
Boa constrictor
Boidae
Anilius scytale
Aniliidae
Amerotyphlops brongersmianus
Amerotyphlops reticulatus
Typhlopidae
Typhlophis squamosus
Liotyphlops beui
Liotyphlops trefauti
Liotyphlops ternetzii
Anomalepididae
Siagonodon acutirostris
Trilepida koppesi
Trilepida fuliginosa
Trilepida macrolepis
Trilepida brasiliensis
Epictia albifrons
Leptotyphlopidae

(continued)
