# Supplementary material for: The Interplay between Environmental Filtering and Spatial Processes in Structuring Communities: The Case of Neotropical Snake Communities
Source: PLoS One. 2015 Jun 10;10(6):e0127959. doi: 10.1371/journal.pone.0127959 (PMC4465701; doi:10.1371/journal.pone.0127959)
Supplement: S1 File — (PDF) [file pone.0127959.s002.pdf]

The interplay between environmental filtering and spatial processes in structuring communities: the case of Neotropical snake communities

Hamanda Cavalheri*, Camila Both and Marcio Martins

*Corresponding author: hbadonac@ucsd.edu

S2 File: Specimens examined

Anilius scytale: Instituto Butantã (IB): 66013, 66466, 66485, 66486, 66488, 66551, 66552, 66554, 67560, 68673. Museu Paraense Emílio Goeldi (MPEG): 16402, 17428, 17456, 23508. ***Apostolepis albicolaris***: IB: 55143. ***Apostolepis ammodites***: IB: 73421, 74131, 74350, 74364, 74373. Coleção Herpetológica da Universidade de Brasília (CHUNB): 35353, 59061, 62376. ***Apostolepis assimilis***: CHUNB: 25357, 25359, 25360, 28950, 28952, 33598. ***Apostolepis dimidiata***: IB: 9507, 23226, 41354, 44471, 46535, 47371, 55707, 65826, 77178, 77765. ***Apostolepis flavotorquata***: CHUNB: 3893, 40751, 40947, 42428, 57321. ***Apostolepis quinquilineata***: MPEG: 2292, 2324, 5325, 11207, 17736. ***Atractus guentheri***: Museu de Zoologia da Universidade Estadual de Santa Cruz (MZUESC): 4867, 5601. ***Atractus latifrons***: IB: 1722, 15057, 15071, 15079, 43399. ***Atractus major***: MPEG: 18231. ***Atractus reticulatus***: IB: 1647, 1650, 3167, 3168, 51347. Coleção Herpetológica da Universidade Federal de Santa Maria (ZUFSM): 162, 528, 630, 788, 927, 1371, 2422. ***Atractus schach***: MPEG: 23451. ***Atractus snethlageae***: MPEG: 17426, 17427, 17539, 21570. ***Atractus torquatus***: MPEG: 17462, 17516. ***Atractus trilineatus***: MPEG: 21315, 21494, 21499. ***Boa constrictor***: MPEG: 19512, 20400, 23986. ***Boiruna maculata***: CHUNB: 3872. IB: 1343, 2567, 19691, 20495, 23430, 23891, 30413, 30672, 42121, 50471, 62801. ZUFSM: 793. ***Bothriopsis bilineata***:

MPEG: 17977. ***Bothropoides jararaca***: Museu de Zoologia da Universidade de São Paulo (MZUSP): 1402, 2253, 2256, 2257, 2260, 2265, 2773, 14209, 14648, 15137. ***Bothropoides lutzi***: CHUNB: 3582, 3583, 3987, 6632. ***Bothropoides marmoratus***: CHUNB: 30838. ***Bothropoides mattogrossensis***: CHUNB: 2905, 24902, 48334. ***Bothropoides neuwiedi***: CHUNB: 25566, 62304. ***Bothropoides pauloensis***: CHUNB: 2907, 3007, 3008, 3580, 3988, 23791. MZUSP: 1990, 1991, 2009, 2222, 2224, 2225, 2226, 2231, 2652, 11652. ***Bothrops atrox***: MPEG: 18755, 18754, 18756, 18807, 19398, 23510, 23512. ***Bothrops jararacussu***: MZUSP: 6528, 10560, 10759, 10760, 11109, 11123. Museu de Zoologia da Universidade Estadual de Campinas (ZUEC): 668, 2922. ***Bothrops leucurus***: MZUESC: 2287, 3636, 6163, 8798, 9699. ***Bothrops pirajai***: MZUESC: 5125. ***Caudisona durissa***: CHUNB: 20434, 20439, 20445, 20450, 20452, 20454, 20458, 20460, 20463, 20465. MZUSP: 2216, 2217, 2220, 5619, 5624, 5625, 5626, 5627, 14476, 17840. ***Chironius bicarinatus***: IB: 18748, 21051, 57985, 64684, 65413, 65415, 71720, 72433, 73803, 77045. ***Chironius carinatus***: MZUESC: 5272. ***Chironius exoletus***: IB: 21019, 22392, 31887, 33529, 46898, 70529. MZUSP: 2565, 3175, 5900, 10564, 13003. ZUEC: 575, 1324, 1367. ***Chironius flavolineatus***: IB: 25297, 25362, 32066, 32774, 34314, 60983, 60987, 60992, 67874. MZUSP: 1894. ***Chironius foveatus***: IB: 21715, 25349, 33459, 71855, 71882, 72185, 73702, 74792. ZUEC: 1062. ***Chironius fuscus***: IB: 40915, 56355, 57194, 58063, 58122, 62288, 73501, 74704, 74706, 74743. MZUSP: 3177, 5779, 5915, 8653, 9640. ***Chironius laevicollis***: IB: 22564, 26186, 26532, 45701, 54574, 55691, 58600, 71561, 72012, 72678, 72707. ***Chironius multiventris***: IB: 17003, 17211, 17285, 17287, 22643, 22970, 27462, 40932, 42848, 42851, 45739. MPEG: 17444, 17515, 17582, 17583. ***Chironius* quadricarinatus**:

CHUNB: 20324, 20329. IB: 17003, 17211, 17284, 17285, 22643, 22970, 27462, 42848, 42851, 45739. ***Chironius scurrulus***: IB: 17636, 23023, 46859, 49608. ***Clelia clelia***: CHUNB: 9673. IB: 24793, 54460. MPEG: 21770, 22387, 23015. ***Clelia plumbea***: IB: 4379, 6471, 15392, 21466, 21496, 27921, 28738, 29319, 30312, 37582, 44629, 50973, 52866. ZUEC: 1325. ***Corallus caninus***: MPEG: 18975, 19453, 19490, 22200. ***Corallus hortulanus***: MPEG: 17385, 17392, 17455, 19386, 19387, 19446, 19451, 20732. ZUEC: 723. ***Dendrophidion dentrophis***: IB: 22144, 71628. ***Dipsas catesbyi***: IB: 42904, 71635. ***Dipsas indica***: IB: 46951, 58379, 53380. ***Dipsas neivai***: IB: 9567, 33328, 55092, 55093, 55094, 58383. ***Dipsas pavonina***: MPEG: 21046. ***Dipsas petersi***: IB: 24636, 25934, 53647, 57090, 57302, 69730, 72221, 73864, 75512, 76737. ***Drepanoides anomalus***: MPEG: 17894, 17931, 19975, 20721, 22392, 22485, 22528, 22932. ***Drymarchon corais***: CHUNB: 3812, 20406. IB: 22917, 42609, 49746, 49838, 49839, 65937, 74144. ***Drymoluber brazili***: IB: 10018, 22515, 24422, 25187, 27896, 32897. ***Drymoluber dichrous***: IB: 2198, 53456. ***Echinanthera cyanopleura***: IB: 877, 5291, 6837, 19491, 23307, 24316, 27923, 33573, 40497, 72030. ***Echinanthera undulata***: IB: 1188, 5037, 13020, 13023, 26745, 26746, 55754, 67165, 68820, 74272, 12900. ***Elapomorphus wuchereri***: Coleção Zoológica Gregório Bondar (CZGB): 831, 4232, 5617. MZUESC: 2810, 8890, 8980. ***Epicrates cenchria***: CHUNB: 3684, 3685, 6638, 20351, 20352, 20353, 24082, 24615, 25593. MPEG: 17486, 23995. MZUSP: 1335, 2662, 4028, 4067. ***Epictia diaplocius***: MPEG: 10168. ***Erythrolamprus aesculapii***: IB: 15031, 29654, 46869, 51899, 52150, 52194, 54196, 55708, 56924, 67503, 71453, 72421, 73874, 74139, 75146. ***Imantodes cenchoa***: MPEG: 17457, 17458, 17839, 18050, 19492, 19494, 19496, 20323. ***Lachesis muta***: MPEG: 12807, 17589, 17763. ***Leptodeira annulata***: MPEG:

17415, 17425, 17513, 19390, 19489, 20317, 20318, 20679, 21065, 21068. ***Leptophis ahaetulla***: IB: 46864, 46887, 46905, 47088, 64352, 64514, 64517, 64537, 64540. ***Liophis almadensis***: CHUNB: 19344, 24244. ZUFSM: 569, 610, 719, 943, 1194, 1377, 1406, 1983, 1984, 2172. ***Liophis flavifrenatus***: ZUFSM: 496, 1136, 1138, 1373, 1376, 1384. ***Liophis jaegeri***: Museu de Ciência e Tecnologia da Pontifícia Universidade Católica do Rio Grande do Sul (MCT): 8368, 11148. MZUSP:, 1115, 1117, 1122, 1127, 2372, 2387, 3289, 4465. ZUEC: 30, 31, 33. ZUFSM: 195, 252, 839, 848, 1243, 1403, 1411, 1432, 2391. ***Liophis meridionalis***: CHUNB: 3753. MZUSP: 1166, 1172, 2452, 2983, 4898. ZUEC: 3081. ***Liophis miliaris***: MCT: 1192, 7884, 10661. MZUSP: 2198, 2483, 2613, 2614, 2616, 2631, 4045, 4451, 4452, 4453, 5040, 8004, 13188, 15054, 17732. ***Liophis paucidens***: CHUNB: 11558, 31335. ***Liophis poecilogyrus***: CHUNB: 3761, 3763, 13843, 20243, 20256, 20259, 20260, 20264, 20267, 20278. MZUSP: 2411, 2423, 3698, 4359, 8350, 13960. ZUEC: 420, 451, 1138, 1591, 3111, 3115, 3143, 3144, 3174, 3179. ZUFSM: 864, 1157, 1286, 1331, 1439, 1495, 1590, 1656, 1715, 1720. ***Liophis reginae***: MPEG: 18227, 21151, 23552, 23623, 23980, 24629, 24630, 24631, 24632. ***Liophis taeniogaster***: MZUESC: 2416, 2901, 5631, 6554. ***Liophis typhlus***: MPEG: 23519. ***Liotyphlops ternetzii***: CHUNB: 3834, 3844, 8518, 10110, 14056, 20344, 23521, 23778, 25350, 27646. ***Mastigodryas bifossatus***: CHUNB: 3828, 20382, 20387, 20388. IB: 25094, 25995, 25996, 25999, 50000, 50001, 50002, 50006, 50007, 50010. ZUFSM: 75, 212, 817, 881, 1153, 1307, 1397, 1433, 1950, 2231. ***Mastigodryas boddaertii***: IB: 34408, 64235, 65198, 65854, 65969, 66188, 66189, 66192, 66251, 73956. ***Micrurus averyi***: MPEG: 17460, 17461, 17555. ***Micrurus corallinus***: MZUSP: 4195. ZUEC: 75, 96, 1039, 1054, 1078, 1338, 1756, 2110, 2642. ***Micrurus frontalis***: CHUNB:

3921, 20314, 20315, 58391. ZUEC: 79, 80, 83, 84, 85, 86, 956, 2188. ***Micrurus hemprichii***: MPEG: 19547. ***Micrurus ibiboboca***: CZGB: 4612, 5356, 5465, 6175, 8174. ***Micrurus spixii***: MPEG: 8452, 8625. ***Micrurus surinamensis***: MPEG: 18243, 20375, 20735, 22060. ***Oxybelis aeneus***: CHUNB: 3714, 3718, 3746, 15484, 52413. IB: 8511, 8661, 8789, 8862, 8863, 8940, 9413, 9731, 46240, 46376, 46862. ***Oxybelis fulgidus***: IB: 3130, 10046, 14786, 43982, 51339, 52189, 64864, 65058, 65816, 76788. ***Oxyrhopus clathratus***: MZUSP: 3479, 4666, 5891, 12440, 15181, 15192, 15215. ZUEC: 1626. ***Oxyrhopus formosus***: MPEG: 10098, 19506, 20861, 22971, 23150, 23202. ***Oxyrhopus guibei***: CHUNB: 3659, 3729, 13753, 13758, 13760, 23823, 24896, 25340, 25356, 28831. MZUSP: 9904, 12050, 12097, 12098, 12106, 12308, 12342, 12442, 12910, 16537. ***Oxyrhopus melanogenys***: MPEG: 17883, 17884, 17930, 17984, 17986. ***Oxyrhopus petola***: IB: 26556, 26580, 27412, 29064, 29065. MPEG: 20367, 20368, 21091. ***Oxyrhopus rhombifer***: CHUNB: 3651, 3667, 3670, 24189, 24195, 24198, 24594, 24607, 24804, 24862, 24900. ZUEC: 3083, 3084, 3086. ZUFSM: 428, 750, 944, 1421, 1610. ***Oxyrhopus trigeminus***: CHUNB: 20301, 20303, 20304. IB: 14200, 14208, 14517, 14588, 22408, 52142, 62626, 69538, 71518. ***Phalotris mertensi***: ZUEC: 1469, 3060. ***Phalotris nasutus***: CHUNB: 23730, 26471, 26483. ***Philodryas aestiva***: CHUNB: 3641, 24238. MCT: 6662, 10656. MZUSP: 207, 208, 215, 2430. ***Philodryas agassizii***: CHUNB: 3671, 3672, 3673, 3677, 3681, 3682, 3690, 3728, 3782. ZUEC: 1108, 2872, 2875, 2881, ZUFSM: 1178, 1458. ***Philodryas argentea***: MPEG: 16862, 17438, 17449, 17484, 17501, 17781, 19459, 20354, 20356, 21098. ***Philodryas nattereri***: CHUNB: 3601, 3612, 13860, 19302, 19304, 19307, 19326, 22086. ***Philodryas olfersi***: MPEG: 16759, 23999. ***Philodryas patagoniensis***: CHUNB: 3781, 19330, 19332, 19335, 19340,

24389, 25355, 28948. MCT: 6943, 7491, 9345, 9911, 9913, 9938, 10527, 10528, 10537, 10657. MZUSP: 235, 239, 1955, 2640, 2645, 4024, 4068, 5519, 12938, 16513. ZUFSM: 331, 342, 967, 971, 1089, 1398, 1404, 1441, 2451, 2538. ***Philodryas psammophidea***: CHUNB: 29405. ***Philodryas viridissima***: MPEG: 17372, 17429. ***Phimophis guerini***: IB: 12245, 22976, 23387, 24116, 26973, 27522, 27985, 37434, 52295, 66025, 66399, 66400, 66401, 66402, 66407, 66411. ***Pseudoboa coronata***: MPEG: 610, 8698, 15713, 21726, 22350. ***Pseudoboa neuwiedii***: IB: 41504. MPEG: 21250, 21410, 22529, 22530, 22531. MZUSP: 5081. ***Pseudoboa nigra***: CHUNB: 3887, 15509, 18322, 20377. IB: 28169, 50604, 52105, 54394, 54528, 56438, 61099. ***Pseustes poecilonotus***: MPEG: 23597, 24204. ***Pseustes sulphureus***: MPEG: 18225, 18648, 19998, 22075. ***Rhachidelus brazili***: CHUNB: 3886, 18344. IB: , 303, 887, 4340, 6620, 7072, 7608, 8356, 9419, 19668, 21103, 24502, 24563, 29462, 32013, 32014, 33780, 46614, 51278, 73454, 76348. ZUEC: 3184. ***Rhinobothryum lentiginosum***: IB: 34470, 73750. MPEG: 22082, 22258. ***Rhinocerophis alternatus***: MCT: 3563, 3603, 5338, 6699. ZUFSM: 278, 311, 667, 763, 950, 1986, 2449. ***Rhinocerophis itapetiningae***: CHUNB: 14342, 19273, 28878, 49445, 49450. ZUEC: 3104, 3105. ***Sibynomorphus mikanii***: MZUSP: 8285, 8288, 8629, 11530, 11736, 12350, 12380, 12746, 13126, 14986. ***Sibynomorphus neuwiedi***: MZUSP: 10803, 12733, 12824, 12833. ZUEC: 1025. ***Sibynomorphus ventrimaculatus***: ZUFSM: 1024, 1118, 1176, 1213, 1235, 1256, 1310, 1361, 1472, 1497. ***Simophis rhinostoma***: CHUNB: 3706, 3708. IB: 4365, 17242, 19420, 26634, 31845, 32760, 52469, 67974, 70341, 75174. MZUSP: 3473. ***Siphlophis cervinus***: IB: 52196. MPEG: 1109, 2672, 16347, 16922, 18479, 18496, 19512, 19743, 21290, 22294, 22639, 22832. MZUSP: 8443, 8444, 9404, 15276. ***Siphlophis compressus***: IB: 50229. MPEG: 17891, 17892, 17893, 23524, 23767,

24197, 24198. ***Siphlophis leucocephalus***: IB: 9141. ***Siphlophis longicaudatus***: IB: 4550, 10485, 26863, 28222, 44259, 49206, 52401, 57387, 60612, 62709, 68053, 73812, 73862, 75226, 75425. ***Siphlophis pulcher***: MZUSP: 295, 2815, 3945, 11258. ZUEC: 1500, 1896, 2146, 2971. ***Siphlophis worontzowi***: IB: 56151. MZUSP: 11345. ***Spilotes pullatus***: IB: 18733, 19469, 22263, 54033, 54891, 67752, 70581, 72597, 75991, 75992. MPEG: 14502, 15200. ***Taeniophallus affinis***: MCP: 6644, 10532. ***Taeniophallus brevirostris***: MPEG: 1569, 17538, 17544, 17574. ***Taeniophallus nicagus***: MPEG: 23709. ***Taeniophallus occipitalis***: CHUNB: 17163, 20367, 24810, 28881. ZUEC: 1658. ***Taeniophallus poecilopogon***: MCT: 6980, 7580. ***Tantilla melanocephala***: CHUNB: 3902, 3902, 10088, 10088. IB: 700, 733, 1039, 2933, 7365, 9730, 42234, 54929, 72222, 75816. MPEG: 9432, 10824, 13374. ***Thamnodynastes hypoconia***: ZUFSM: 1593, 2455. ***Thamnodynastes strigatus***: MZUSP: 2362, 4360, 4575, 4909, 12445, 14008, 16486, 16504. ZUFSM: 167, 714. ***Tomodon dorsatus***: MCT: 4116, 4193. MZUSP: 266, 4082, 4519, 4578, 12206, 13186, 14642, 16530, 16539, 16558. ***Tricheilostoma fuliginosum***: CHUNB: 40738. MZUSP: 17671, 17854. ***Tricheilostoma koppesi***: MZUSP: 14976, 14977. ZUEC: 577. ***Tropidodryas serra***: MZUSP: 10240, 12443. ZUEC: 1580, 2148. ***Typhlops reticulatus***: MPEG: 17738, 17958, 17992, 17993, 17994, 20349, 23526, 23967. ***Xenodon dorbignyi***: ZUFSM: 743, 776, 1181, 2526. ***Xenodon merremii***: CHUNB: 20566, 20569, 20576, 20580, 20586, 20588, 25598. MZUSP: 1775, 2798, 2967, 3506, 3507, 3523, 3675, 4075, 4319, 4574, 8554. ZUFSM: 56, 623, 624, 806, 837, 923, 1040, 1535, 1880, 2213. ***Xenodon nattereri***: CHUNB: 3793. ZUEC: 3106, 3142. ***Xenodon neuwiedii***: MZUSP: 973, 975, 4576, 12444, 12449, 12918, 12919, 12943, 12979, 13164, 16485. ***Xenodon rhabdocephalus***: MPEG: 4125, 5782, 14210, 17818,

17830, 17934, 22105, 22107. ***Xenodon severus***: MPEG: 17759, 24232. ***Xenopholis scalaris***: MPEG: 17471, 17565, 17935, 19568, 20370, 20371, 20963.
