# Supplementary material for: The Interplay between Environmental Filtering and Spatial Processes in Structuring Communities: The Case of Neotropical Snake Communities
Source: PLoS One. 2015 Jun 10;10(6):e0127959. doi: 10.1371/journal.pone.0127959 (PMC4465701; doi:10.1371/journal.pone.0127959)
Supplement: S2 File — (PDF) [file pone.0127959.s003.pdf]

The interplay between environmental filtering and spatial processes in structuring communities: the case of Neotropical snake communities

Hamanda Cavalheri*, Camila Both and Marcio Martins

*Corresponding author: hbadonac@ucsd.edu

S5 File: Construction of snake topology

The following studies were used to construct the topology. The phylogenies of [1, 2] were used for the relative placement of snake families, subfamilies and tribes. Families Leptotyphlopidae, Typhlopidae, and Anomalepididae were repositioned using the phylogeny of [3]. The phylogenies of [4, 5] were used for relationships within Boidae. Within Viperidae, the phylogeny of [6] was used to determine the relationship of *Bothrops* species (expect for *Bothrops lutzi*, which was repositioned using [7]), whereas [8] was used for the relationships of *Crotalus* and *Lachesis*. The relationships of species of Elapidae were determined using the phylogeny of [9]. The overall relationships within Colubridae follow [1], but, within Colubridae, the relative positioning of *Chironius* species was determined according to [10]. Within Dipsadinae, Elapomorphini species had their positions determined according to [11] (*Phalotris bilineatus*), [12] (*Phalotris multipuctatus*), [13] (*Apostolepis* species), [14] (*Apostolepis assimilis* and *A. dimidiata*), and [15] (*Phalotris concolor*). Dipsadini species, specifically the genera *Atractus* and *Dipsas,* were replaced according P. Passos, (pers. com.), and [16, 17], respectively. Placement of Echinantherini species was determined using the phylogeny of [18], whereas placement of Philodryadini, Xenodontini, and Pseudoboini was determined using the phylogeny of [19].

References

1. Pyron RA, Burbrink FT, Colli GR, Oca ANM, Vitt LJ, Kuczynski CA, Wiens JJ (2011) The phylogeny of advanced snakes (Colubroidea), with discovery of a new subfamily and comparison of support methods of likelihood trees. Mol Phylogenet Evol. 58: 329-342. doi: 10.1016/j.ympev.2010.11.006

2. Zaher H, Grazziotin FG, Cadle JE, Murphy RW, Moura-Leite JC, Bonatto SL (2009) Molecular phylogeny of advanced snakes (Serpentes, Caenophidia) with an emphasis on South American Xenodontines: a revised classification and descriptions of new taxa. Pap Avulsos Zool. 49 (11): 115-153. doi: 10.1590/S0031-10492009001100001

3. Lee MSY, Scanlon JD (2002) Snake phylogeny based on osteology, soft anatomy and ecology. Biol Rev. 77: 333-401. doi: 10.1017}S1464793102005924

4. Kluge AG (1991) Boinae snake phylogeny and research cycles. Misc Publ - Mus. Zool, Univ Mich. 158: 1-58.

5. Burbrink FT (2005) Inferring the phylogenetic position of *Boa constrictor* among the Boinae. Mol Phylogenet Evol 34: 167-180. doi: 10.1016/j.ympev.2004.08.017

6. Fenwick AM, Gutberlet RL, Evans JA, Parkinson CL (2009) Morphological and molecular evidence for phylogeny and classification of South American pitvipers, genera *Bothrops, Bothriopsis,* and *Bothrocophias* (Serpentes: Viperidae). Zool J Linnean Soc 156: 617-640. doi: 10.1111/j.1096-3642.2008.00495.x

7. Silva VX, Rodrigues MT (2008) Taxonomic revision of the *Bothrops neuwiedi* complex (Serpentes, Viperidae) with description of a new species. Phyllomedusa 7 (1): 45-90. doi: 10.1590/S1519-13972008000100005

8. Wüster W, Peppin L, Pook CE, Walker DE (2008) A nesting of vipers: phylogeny and historical biogeography of the Viperidae (Squamata: Serpentes). Mol Phylogenet Evol 49: 445-459. doi: 10.1016/j.ympev.2008.08.019

9. Silva NJ, Sites JW (2001) Phylogeny of South America triad coral snakes (Elapidae: *Micrurus*) based on molecular characters. Herpetologica 57 (1): 1-22.

10. Klaczko J (2007) Filogenia do gênero *Chironius* (Serpentes, Colubridae) baseada em dados morfológicos e moleculares. PhD dissertation, Universidade de São Paulo. Available: http://www.teses.usp.br/teses/disponivei/41/41133/tde-26042007-144952/pt-br.php. Accessed 20 June 2012.

11. Ferrarezzi H (1993) Nota sobre o gênero *Phalotris* com revisão do grupo *nasutus* e descrição de três novas espécies (Serpentes, Colubridae, Xenodontinae). Mem Inst Butantan 55 (1): 21-38.

12. Puorto G, Ferrarezzi H (1993) Uma nova espécie de *Phalotris* Cope, 1862, com comentários sobre o grupo *bilineatus* (Serpentes: Colubridae: Xenodontinae). Mem Inst Butantan 55 (1): 39-46.

13. Ferrarezzi H, Barbo FE, Albuquerque CE (2005) Phylogenetic relationships of a new species of *Apostolepis* from Brazilian Cerrado with notes on the *assimilis* group (Serpentes: Colubridae: Xenodontinae: Elapomorphini). Pap Avulsos Zool. 45 (16): 215-229. doi: 10.1590/S0031-10492005001600001

14. Giraudo AR, Scrocchi GJ (1998) A new species of *Apostolepis* (Serpentes: Colubridae) and comments on the genus in Argentina. Herpetologica 54 (4): 470-476.

15. Cacciali P, Carreira S, Scott N (2007) Redescription of *Phalotris nigrilatus* Ferrarezzi, 1993 (Serpentes: Colubridae: Xenodontinae). Herpetologica 63 (4): 552-559. doi: 10.1655/00180831(2007)63[552:ROPNFS] 2.0.CO;2

16. Passos P, Fernandes R, Bérnils RS, Moura-Leite JC (2010) Taxonomic revision of the Brazilian Atlantic Forest *Atractus* (Reptilia: Serpentes: Dipsadidae). Zootaxa 2364: 1-63.

17. Harvey MB (2008) New and poorly known *Dipsas* (Serpentes: Colubridae) from northern South America. Herpetologica 64 (4): 422-451. doi: 10.1655/07-068R1.1

18. Santos Jr. AP (2009) Revisão taxonômica do grupo de *Taeniophallus occipitalis* e o relacionamento filogenético da Tribo Echinantherini (Serpentes, Dipsadidae, Xenodontinae). Phd dissertation, Pontifícia Universidade Católica do Rio Grande do Sul. Available: http://tede.pucrs.br/tde_busca/ arquivo.php?codArquivo=2573. Accessed 20 June 2012.

19. Grazziotin FG, Zaher H, Murphy RW, Scrocchi G, Benavides MA, Zhang YP, Bonatto SL (2012) Molecular phylogeny of the New World Dipsadidae (Serpentes: Colubroidea): a reappraisal. Cladistics 28: 437–459. doi: 10.1111/j.1096-0031.2012.00393.x
