# Supplementary material for: The Interplay between Environmental Filtering and Spatial Processes in Structuring Communities: The Case of Neotropical Snake Communities
Source: PLoS One. 2015 Jun 10;10(6):e0127959. doi: 10.1371/journal.pone.0127959 (PMC4465701; doi:10.1371/journal.pone.0127959)
Supplement: S1 Table — (PDF) [file pone.0127959.s004.pdf]

The interplay between environmental filtering and spatial processes in structuring communities: the case of Neotropical snake communities

Hamanda Cavalheri*, Camila Both and Marcio Martins

*Corresponding author: hbadonac@ucsd.edu

S1 Table: Community composition

**FAMILY**<br>SUBFAMILY<br>*Species*	Community											
	AM 1	AM 2	AT 1	AT 2	AT 3	AT 4	CE 1	CE 2	CE 3	CE 4	CA 1	CA 2
**ANILIIDAE**												
Anilius scytale	X	X										
**ANOMALEPIDIDAE**												
Liotyphlops beui					X							
Liotyphlops ternetzii									X			
Liotyphlops trefauti			X									
Typhlophis squamosus	X											

**FAMILY**<br>SUBFAMILY<br>*Species*	Community											
	AM 1	AM 2	AT 1	AT 2	AT 3	AT 4	CE 1	CE 2	CE 3	CE 4	CA 1	CA 2
**BOIDAE**												
Boa constrictor	X	X	X				X	X				
Corallus caninus	X	X										
Corallus cropanii				X								
Corallus hortulanus	X	X	X	X								
Epicrates assisi							X					
Epicrates cenchria	X	X	X						X	X		
Epicrates crassus								X				
**COLUBRIDAE**												
Chironius bicarinatus			X	X	X	X						
Chironius carinatus			X									
Chironius exoletus		X	X	X			X					
Chironius flavolineatus							X	X		X		
Chironius foveatus			X	X								
Chironius fuscus	X		X	X								
Chironius laevicollis			X	X								

**FAMILY**<br>SUBFAMILY<br>*Species*	Community											
	AM 1	AM 2	AT 1	AT 2	AT 3	AT 4	CE 1	CE 2	CE 3	CE 4	CA 1	CA 2
Chironius multiventris	X											
Chironius quadricarinatus									X			
Chironius scurrulus	X	X										
Dendrophidion dentrophis	X	X										
Drymarchon corais		X	X						X			
Drymobius rhombifer		X										
Drymoluber brazili									X			
Drymoluber dichrous	X	X	X									
Leptophis ahaetulla	X	X	X				X					
Mastigodryas bifossatus			X				X	X	X			X
Mastigodryas boddaerti	X	X					X					
Oxybelis aeneus	X		X					X	X			
Oxybelis fulgidus	X	X										
Rhinobothryum lentiginosum	X	X										
Spilotes poecilonotus	X	X										
Spilotes pullatus	X	X	X	X			X					

**FAMILY**<br>SUBFAMILY<br>*Species*	Community											
	AM 1	AM 2	AT 1	AT 2	AT 3	AT 4	CE 1	CE 2	CE 3	CE 4	CA 1	CA 2
Spilotes sulphureus	X		X									
Simophis rhinostoma									X	X		
Tantilla melanocephala	X	X	X				X	X	X	X		X
**DIPSADIDAE**												
DIPSADINAE												
Atractus alphonsehogei	X											
Atractus guentheri			X									
Atractus latifrons	X	X										
Atractus major	X											
Atractus poeppigi	X											
Atractus reticulatus												X
Atractus schach	X											
Atractus snethlageae	X											
Atractus torquatus	X											
Atractus trilineatus	X											
Dipsas catesbyi		X	X									

**FAMILY**<br>SUBFAMILY<br>*Species*	Community										
	AM 1	AM 2	AT 1	AT 2	AT 3	AT 4	CE 1	CE 2	CE 3	CE 4	CA 1	CA 2
Dipsas incerta				X								
Dipsas indica	X	X	X									
Dipsas indica petersi			X	X								
Dipsas pavonina	X	X										
Dipsas variegata			X									
Imantodes cenchoa	X	X	X	X								
Leptodeira annulata	X	X	X									
Ninia hudsoni		X										
Sibynomorphus mikanii										X		
Sibynomorphus neuwiedi			X	X	X							
Sibynomorphus ventrimaculatus												X
XENODONTINAE												
Apostolepis albicolaris									X			
Apostolepis ammodites									X			
Apostolepis assimilis									X			
Apostolepis cearensis							X					

**FAMILY**<br>SUBFAMILY<br>*Species*	Community											
	AM 1	AM 2	AT 1	AT 2	AT 3	AT 4	CE 1	CE 2	CE 3	CE 4	CA 1	CA 2
Apostolepis dimidiata										X		
Apostolepis flavotorquata									X			
Apostolepis nelsonjorgei								X				
Apostolepis polylepis							X					
Apostolepis quinquelineata	X											
Boiruna maculata					X				X	X		X
Boiruna sertaneja							X	X				
Clelia clelia	X											
Clelia plumbea		X	X	X								
Coronelaps lepidus			X									
Drepanoides anomalus	X	X										
Echinanthera cyanopleura				X	X	X						
Echinanthera undulata				X								
Elapomorphus wuchereri			X									
Erythrolamprus aesculapii	X		X	X								
Erythrolamprus almadensis								X	X			X

**FAMILY**<br>SUBFAMILY<br>*Species*	Community											
	AM 1	AM 2	AT 1	AT 2	AT 3	AT 4	CE 1	CE 2	CE 3	CE 4	CA 1	CA 2
Erythrolamprus breviceps	X											
Erythrolamprus cobella			X									
Erythrolamprus jaegeri										X	X	X
Erythrolamprus maryellenae								X				
Erythrolamprus poecilogyrus			X		X	X	X		X	X		X
Erythrolamprus pygmaeus	X											
Erythrolamprus reginae	X	X	X									
Erythrolamprus taeniogaster			X									
Erythrolamprus typhlus	X											
Lygophis flavifrenatus												X
Lygophis meridionalis								X	X	X		
Lygophis miliaris			X	X	X	X					X	
Lygophis paucidens								X	X			
Oxyrhopus clathratus				X	X							
Oxyrhopus formosus			X									
Oxyrhopus guibei			X					X	X	X		

**FAMILY**<br>SUBFAMILY<br>*Species*	Community											
	AM 1	AM 2	AT 1	AT 2	AT 3	AT 4	CE 1	CE 2	CE 3	CE 4	CA 1	CA 2
Oxyrhopus melanogenys		X										
Oxyrhopus occipitalis	X											
Oxyrhopus petolarius digitalis		X	X									
Oxyrhopus rhombifer					X	X	X		X	X		X
Oxyrhopus trigeminus								X	X			
Phalotris labiomaculatus								X				
Phalotris lativittatus										X		
Phalotris mertensi										X		
Phalotris multipunctatus										X		
Phalotris nasutus									X			
Philodryas aestiva						X			X	X	X	
Philodryas agassizii									X	X		X
Philodryas argentea	X	X										
Philodryas arnaldoi											X	
Philodryas livida										X		
Philodryas nattereri							X	X	X			

**FAMILY**<br>SUBFAMILY<br>*Species*	Community											
	AM 1	AM 2	AT 1	AT 2	AT 3	AT 4	CE 1	CE 2	CE 3	CE 4	CA 1	CA 2
Philodryas olfersii		X	X		X		X					
Philodryas patagoniensis						X			X	X	X	X
Philodryas psammophidea									X			
Philodryas viridissima	X											
Phimophis guerini								X	X	X		
Pseudoboa coronata	X											
Pseudoboa martinsi	X											
Pseudoboa neuwiedii	X											
Pseudoboa nigra			X						X			
Psomophis joberti							X	X				
Rhachidelus brazili									X	X		
Rodriguesophis iglesiasi							X	X				
Siphlophis cervinus	X											
Siphlophis compressus	X	X	X									
Siphlophis leucocephalus			X									
Siphlophis longicaudatus				X								

**FAMILY**<br>SUBFAMILY<br>*Species*	Community											
	AM 1	AM 2	AT 1	AT 2	AT 3	AT 4	CE 1	CE 2	CE 3	CE 4	CA 1	CA 2
Siphlophis pulcher			X	X								
Siphlophis worontzowi		X										
Taeniophallus affinis					X						X	
Taeniophallus bilineatus					X							
Taeniophallus brevirostris	X											
Taeniophallus nicagus	X											
Taeniophallus occipitalis									X	X		
Taeniophallus poecilopogon						X					X	
Thamnodynastes hypoconia					X							X
Thamnodynastes strigatus				X	X	X						X
Tomodon dorsatus				X	X	X					X	
Tropidodryas serra			X	X								
Uromacerina ricardinii				X								
Xenodon dorbignyi												X
Xenodon merremii					X		X	X	X	X		X
Xenodon nattereri									X	X		

**FAMILY**<br>SUBFAMILY<br>*Species*	Community<br>AM 1	AM 2	AT 1	AT 2	AT 3	AT 4	CE 1	CE 2	CE 3	CE 4	CA 1	CA 2
Xenodon neuwiedii				X	X							
Xenodon rabdocephalus	X	X	X									
Xenodon severus		X										
Xenopholis scalaris	X	X	X									
**ELAPIDAE**												
Micrurus altirostris					X	X						
Micrurus averyi	X											
Micrurus brasiliensis								X				
Micrurus corallinus			X	X								
Micrurus frontalis									X	X		X
Micrurus hemprichii	X	X										
Micrurus ibiboboca			X				X					
Micrurus lemniscatus	X											
Micrurus spixii	X	X										
Micrurus surinamensis	X	X										

FAMILY<br>SUBFAMILY<br>*Species*	Community											
	AM 1	AM 2	AT 1	AT 2	AT 3	AT 4	CE 1	CE 2	CE 3	CE 4	CA 1	CA 2
**LEPTOTYPHLOPIDAE**												
Epictia albifrons	X											
Siagonodon acutirostris								X				
Trilepida brasiliensis							X	X				
Trilepida fuliginosa									X			
Trilepida koppesi										X		
Trilepida macrolepis		X										
**TYPHLOPIDAE**												
Amerotyphlops brongersmianus			X					X				
Amerotyphlops reticulatus	X	X										
**VIPERIDAE**												
Bothrops alternatus			X			X					X	X
Bothrops atrox	X	X										
Bothrops bilineatus		X	X									
Bothrops itapetiningae									X	X		
Bothrops jararaca			X	X	X							

**FAMILY**												
SUBFAMILY	Community											
Species	AM 1	AM 2	AT 1	AT 2	AT 3	AT 4	CE 1	CE 2	CE 3	CE 4	CA 1	CA 2
Bothrops jararacussu				X								
Bothrops leucurus			X									
Bothrops lutzi							X	X	X			
Bothrops marmoratus									X			
Bothrops mattogrossensis									X			
Bothrops moojeni							X	X				
Bothrops neuwiedi					X	X			X			
Bothrops pauloensis									X	X		
Bothrops pirajai			X									
Crotalus durissus							X	X	X	X		
Lachesis muta	X	X	X									
