# Supplementary material for: The Interplay between Environmental Filtering and Spatial Processes in Structuring Communities: The Case of Neotropical Snake Communities
Source: PLoS One. 2015 Jun 10;10(6):e0127959. doi: 10.1371/journal.pone.0127959 (PMC4465701; doi:10.1371/journal.pone.0127959)
Supplement: S2 Table — (PDF) [file pone.0127959.s005.pdf]

The interplay between environmental filtering and spatial processes in structuring communities: the case of Neotropical snake communities

Hamanda Cavalheri*, Camila Both and Marcio Martins

*Corresponding author: hbadonac@ucsd.edu

S3 Table: Species traits

All measurements are average values for each trait and each species represented by two or more individuals in millimeters, except VS and SS. SVL = Snout-vent length; TL = tail length; HL = head length; HW = head width; HH = head height; CAM = circumference around midbody; VS = ventral scales count; SS = subcaudal scales count.

**FAMILY**<br>SUBFAMILY<br>*Species*	Morphological traits							
	SVL	TL	HL	HW	HH	CAM	VS	SS
**ANILIIDAE**								
Anilius scytale	603.71	28	19.01	11.17	9.04	42.57	228.43	13.71
**ANOMALEPIDIDAE**								
Liotyphlops ternetzii	266.7	4.56	5.33	3.11	2.58	15.6	317.3	12.4
**BOIDAE**								

**FAMILY**<br>SUBFAMILY<br>*Species*	Morphological traits							
	SVL	TL	HL	HW	HH	CAM	VS	SS
Boa constrictor	905	89.67	41.87	26.47	16.03	110	228.67	49.67
Corallus caninus	1019.25	144.25	45.35	26.5	20.1	92	204.75	67.75
Corallus hortulanus	1248.43	303.86	37.71	23.51	17.11	86.57	265.64	112
Coralus hortulanus	1034	263	35.4	22.9	12.4	80	264	109
Epicrates cenchria	614.12	72.71	31.2	18.32	12.82	80.08	218.75	42.33
**COLUBRIDAE**								
Chironius bicarinatus	799.7	469	27.31	14.41	10.43	54.7	162.6	144.6
Chironius carinatus	1287	545	46	26.95	18.65	90	154	120
Chironius exoletus	755.67	426.83	30.57	15.65	11.89	52.94	151.17	135.06
Chironius flavolineatus	699.5	419.3	24.29	11.12	8.1	47.3	159.1	134.9
Chironius foveatus	1104.89	641.78	36.28	18.03	12.89	69.44	174.33	155.67
Chironius fuscus	725.93	409.33	26.57	14.02	10.33	60.4	147.67	124.8
Chironius laevicollis	1220.22	539.44	49.42	25.03	17.56	94.22	157.89	108.56
Chironius multiventris	1349.25	725.5	39.98	20.07	14.3	66.25	193	184.75
Chironius quadricarinatus	625.17	348.08	24.34	11.12	8.16	44.5	153.75	118.33
Chironius scurrulus	1283.75	587	51.33	27.73	18.43	74.22	157.5	108.25
Dendrophidion dendrophis	520	448.5	19.65	10.05	7.35	40	154	163

**FAMILY**<br>SUBFAMILY<br>*Species*	Morphological traits							
	SVL	TL	HL	HW	HH	CAM	VS	SS
Drymarchon corais	1578.2	310.53	53.25	32.06	24.02	121.33	211.13	73.87
Drymoluber brazili	853	364	27.9	13.4	9.7	57	197	110
Drymoluber dichrous	756	89.67	27.43	14.67	11.33	58.67	169.67	24.33
Leptophis ahaetulla	649.93	401.14	21.64	10.54	6.86	40.57	165.07	152
Mastigodryas bifossatus	1294.43	420.39	50.26	27.18	19.14	103.89	180.61	89.18
Mastigodryas boddaertii	836.5	297.8	29.87	14.23	10.66	53.1	197.3	103.3
Oxybelis aeneus	735.48	453.71	26.42	7.82	7.75	34.81	192.1	165.29
Oxybelis fulgidus	1198.3	546.8	46.36	19.63	14.86	61.5	209.8	151.7
Rhinobothryum lentiginosum	879.75	220.25	25.23	16	10.7	52.5	249	105.5
Spilotes poecilonotus	1028	382.5	38.9	20	13.85	80	298.5	120
Spilotes pullatus	1541.58	467.5	46.09	27.18	20.02	103.33	213	108.67
Spilotes sulphureus	883	292.5	33.92	17.77	13.7	59	214.25	127
Simophis rhinostoma	589.77	154.62	22.02	11.72	8.67	44.46	182	66.15
Tantilla melanocephala	250.24	69.65	9.26	4.74	3.75	22.65	150.82	49.76
**DIPSADIDAE**								
DIPSADINAE								
Atractus guentheri	429.5	45	20.62	15	8.97	39	145	23

**FAMILY**<br>SUBFAMILY<br>*Species*	Morphological traits							
	SVL	TL	HL	HW	HH	CAM	VS	SS
Atractus latifrons	444	59.6	14.1	8.06	7.26	35	162.6	30.8
Atractus major	443	58	16.3	8.1	6.6	40	160	27
Atractus reticulatus	325.67	40.83	12.07	6.17	5.31	33.42	154.25	23.75
Atractus schach	64	25	9.2	5.3	3.4	20	135	22
Atractus snethlageae	364	35.75	13.22	7.9	5.4	33.75	164	22.25
Atractus torquatus	444	64.5	16.45	8.05	6.35	35	164.5	36.5
Atractus trilineatus	258.33	21.67	8.97	4.93	3.93	29	145	13.33
Dipsas catesbyi	383	137	13.9	7.05	5.05	27	179.5	92
Dipsas indica	506	175.33	16.3	11.5	7.9	34	182.67	96.33
Dipsas indica petersi	663.7	227.6	30.32	17.61	13.13	48.7	184.4	93.8
Dipsas pavonina	202	87	9.2	5.3	4.8	21	197	86
Dipsas variegata	580.17	154	22.67	12.02	9.45	40.5	183	74.5
Imantodes cenchoa	702.09	292.73	13.59	8.65	5.87	26.91	246.45	138.82
Leptodeira annulata	492.8	157.87	18.45	11.4	7.22	38.53	188.8	81.4
Sibynomorphus mikani	412.7	79.1	15.36	8.89	6.76	36.8	164.8	45.3
Sibynomorphus neuwiedi	492.4	123.4	17.64	10.18	7.22	39.8	170.2	64.8
Sibynomorphus ventrimaculatus	367.2	80.8	15.32	7.9	6.73	35	152.9	40.1

FAMILY								
SUBFAMILY	Morphological traits							
Species	SVL	TL	HL	HW	HH	CAM	VS	SS
XENODONTINAE								
Apostolepis albicolaris	258	33	6.8	4	2.8	17	220	31
Apostolepis ammodites	521.12	44	12.84	6.96	5.45	30.5	244.12	27.62
Apostolepis assimilis	249.67	26.83	7.38	3.7	2.6	16	226	21.17
Apostolepis dimidiata	492.2	43.5	11.22	5.22	4.02	24.3	250.5	28
Apostolepis flavotorquata	476.2	43.4	11.8	6.2	4.56	26.6	253.2	27.8
Apostolepis quinquilineata	472.6	49.6	12.38	7.66	4.52	29.6	225.4	27.2
Boiruna maculata	1255.85	201.62	39.07	23.75	16.92	89.46	223.85	57
Clelia clelia	1477.17	302.83	43.05	28.38	17.98	107.33	235	75.83
Clelia plumbea	1730.22	333.11	51.23	30.75	20	109.72	232.83	89.17
Drepanoides anomalus	398	114	14.6	8.23	6.2	29	171.67	65
Echinanthera cyanopleura	499	223.6	19.63	9.4	6.56	37.5	153.1	92.5
Echinanthera undulata	432.73	181.55	17.78	8.62	6.55	34.45	147	83.64
Elapomorphus wuchereri	820.33	87.17	29.1	16.94	13.13	56.5	182.17	33
Erythrolamprus aesculapii	701.07	93.4	22	12.78	9.61	46.47	189.47	43.13
Erythrolamprus almadensis	395.83	99.83	17.91	9.83	7.18	37.5	152.92	59.17
Erythrolamprus jaegeri	384.62	103.05	15.8	8.31	6.5	37.19	157.86	56.81

**FAMILY**<br>SUBFAMILY<br>*Species*	Morphological traits							
	SVL	TL	HL	HW	HH	CAM	VS	SS
Erythrolamprus poecilogyrus	461.49	103.6	22.27	13.1	9	43.83	157.91	53.11
Erythrolamprus reginae	378.22	105.33	16.73	9.14	6.07	35.44	142.56	51.33
Erythrolamprus taeniogaster	547.75	108.25	24.91	13.91	11.1	52.5	152.25	50
Erythrolamprus typhlus	367	77	18.8	10.5	7.6	44	139	42
Lygophis flavifrenatus	505.17	188.67	18.28	9.38	7.1	38.5	163.67	78.67
Lygophis meridionalis	537.14	191.43	19.45	9	6.99	37.86	170.57	81.29
Lygophis miliaris	623	124.72	26.16	15.7	11.81	54.78	156.61	51.5
Lygophis paucidens	365	115.5	15.4	6.9	5.35	31	169	74
Oxyrhopus clathratus	865.25	179.12	25.6	13.85	10.62	67.5	204.38	67.5
Oxyrhopus formosus	764.33	176	22.96	11.66	7.98	49.33	198.5	74.33
Oxyrhopus guibei	577.37	132.9	19.01	10.02	7.41	44.67	201.87	67.53
Oxyrhopus melanogenys	531.4	120.2	17.5	8.92	6.06	39.4	201.4	59.6
Oxyrhopus petolarius digitalis	666.25	174	21.89	11.11	7.85	45.75	199.75	81.62
Oxyrhopus rhombifer	478.68	100.53	17.24	9.51	6.81	42.26	188.63	59.21
Oxyrhopus trigeminus	626.75	127.92	20.21	10.63	7.8	42.75	206.83	66.67
Phalotris mertensi	536.5	48.5	13.35	8.1	5.95	30.5	218.5	26.5
Phalotris nasutus	488.67	46.33	14.53	8.5	6.03	34	193.67	26.67

**FAMILY**<br>SUBFAMILY<br>*Species*	Morphological traits							
	SVL	TL	HL	HW	HH	CAM	VS	SS
Philodryas aestiva	704.12	274.38	23.34	11.35	8.78	49.62	195.38	112.5
Philodryas agassizii	291.4	87.53	14.55	7.49	6.11	31.13	136.8	54.07
Philodryas argentea	700.8	439	23.41	8.04	7.23	35	207.8	178.4
Philodryas nattereri	768.75	297.75	30.45	17.3	11.82	63	208.62	107.5
Philodryas olfersi	717.33	263.33	24.77	12.97	9.8	52.33	194.67	104.67
Philodryas patagoniensis	854.63	279.24	31.86	16.68	13.38	65.63	188.03	95.47
Philodryas psammophidea	392	125	18.8	10.4	8.6	40	156	65
Philodryas viridissima	542	195.5	20.25	11.3	7.75	38	216	112.5
Phimophis guerini	792.88	155.62	22.88	13.34	9.38	63.19	209.38	66
Pseudoboa coronata	723	200	25.5	14.54	9.52	59.4	190.6	80.8
Pseudoboa neuwiedii	774	172.29	25.46	13.6	8.82	56.86	193.29	65.43
Pseudoboa nigra	640.64	196.18	23.67	14.4	9.41	59.91	199.82	82.45
Rhachidelus brazili	950.22	205.3	39.4	22.26	13.28	98.87	186.96	72.17
Siphlophis cervinus	724.76	210.18	19.59	11.54	7.1	36.71	251.53	111.06
Siphlophis compressus	702.88	189.88	19.24	12.29	7.55	111.5	248.62	106.12
Siphlophis leucocephalus	514	147	17.57	10.46	5.66	34	232	109
Siphlophis longicaudatus	733.87	222.47	20.74	12.06	7.09	42.87	221.87	100.73

**FAMILY**								
SUBFAMILY	Morphological traits							
Species	SVL	TL	HL	HW	HH	CAM	VS	SS
Siphlophis pulcher	619.12	172.12	17.18	10.9	7.36	43	234.38	96.12
Siphlophis worontzowi	522	121	16.65	8.5	6.73	31.5	228	108
Taeniophallus affinis	386	102.5	13.95	7.65	5.35	29.5	180.5	61
Taeniophallus brevirostris	253	57.75	11.07	6.53	4.7	26.75	142.5	35.5
Taeniophallus nicagus	356	93	13.1	7.1	5	31	179	51
Taeniophallus occipitalis	355.2	115.4	13.02	6.02	4.7	24	183.2	79.4
Taeniophallus poecilopogon	309.5	94	13.05	6.1	3.3	27	152.5	60
Thamnodynastes hypoconia	369	119.5	18.45	8.75	7.3	41	142	63.5
Thamnodynastes strigatus	506.4	129.6	27.11	16.32	11.85	61.1	140.3	56.1
Tomodon dorsatus	520.92	146.08	22.49	12.63	10.01	56.17	145.5	64.08
Tropidodryas serra	986.5	218.5	34.92	20.48	13.7	66.25	220.5	87.75
Xenodon dorbignyi	499	65.75	21.73	13.25	10.6	53.75	130.25	32
Xenodon merremii	860.86	123.54	44.54	29.32	19.84	93.79	147.75	37.36
Xenodon nattereri	323	51	15.37	8.5	7.27	34.33	145.67	28.67
Xenodon neuwiedii	629.73	116.27	27.67	17.05	11.57	57.45	164.91	57.82
Xenodon rabdocephalus	565	93.88	27.02	17.93	10.88	57.75	143.5	38.38
Xenodon severus	437.5	65.5	27.65	20.55	12.65	57.5	127.5	33.5

**FAMILY**<br>SUBFAMILY<br>*Species*	Morphological traits<br>SVL	 TL	 HL	 HW	 HH	 CAM	 VS	 SS
Xenopholis scalaris	251.8	49	10.47	5.64	3.95	27.4	134.9	28.9
**ELAPIDAE**								
Micrurus averyi	478.67	46	13.23	7.43	5.3	28.33	208.33	24.67
Micrurus corallinus	698.2	89.7	19.08	11.41	8.45	42.2	213.8	30.8
Micrurus frontalis	787.33	47.5	19.85	11.1	7.6	41.25	227.75	20.33
Micrurus hemprichii	436	48	13.8	7.9	5.5	30	173	24
Micrurus ibiboboca	801.8	146.6	23.03	13.28	10.37	47.6	213.6	20.4
Micrurus spixii	452.5	32.5	14	7.9	5.55	30.5	207.5	19.5
Micrurus surinamensis	531.75	66.25	22.6	15.18	9.15	49.5	163.75	28.75
**LEPTOTYPHLOPIDAE**								
Epictia albifrons	183	18	4.8	2.9	2.4	20	180	17
Trilepida fuliginosa	163.33	19.33	7.17	3.73	2.93	21	175.67	16.67
Trilepida koppesi	222	20.67	7.7	4.13	3.6	25.33	172.67	12.67
**TYPHLOPIDAE**								
Amerotyphlops reticulatus	320.88	11.5	11.35	8.18	5.69	39.12	216	8.5
**VIPERIDAE**								
Bothrops alternatus	1002.91	102.27	47.11	31.15	18.98	119.64	176	36.27

**FAMILY**<br>SUBFAMILY<br>*Species*	Morphological traits							
	SVL	TL	HL	HW	HH	CAM	VS	SS
Bothrops atrox	926.86	127.29	41.96	26.94	16.84	85.43	199.29	56.29
Bothrops bilineatus	649	95	27.2	14.5	10	44	214	56
Bothrops itapetiningae	446.86	48.71	25.91	16.04	11.33	58.29	149.57	28.43
Bothrops jararaca	995.2	132.7	40.32	26.07	17.16	82.6	199.8	59.7
Bothrops jararacussu	1242.75	149.75	61.22	45.29	25.16	147	177.12	51.38
Bothrops leucurus	1022.4	142.2	45.51	29.74	18.28	92.8	199.6	60.4
Bothrops lutzi	582.75	78.25	29.23	18.38	11.4	59.25	169.75	41.75
Bothrops marmoratus	621	65	28.9	18.7	10.9	67	173	34
Bothrops mattogrossensis	558.33	78	27.9	18.33	11.5	53	171.33	45.33
Bothrops neuwiedi	535.5	71.5	26.55	17.8	12.65	66	168.5	46.5
Bothrops pauloensis	546.69	66.44	27.17	16.61	11.26	54.25	172.44	40.94
Bothrops pirajai	892	132	50.5	36.6	22.5	124	158	58
Crotalus durissus	879.95	69.25	42.87	28.8	19.69	124.9	175.5	23.55
Lachesis muta	1576	146	62.3	41.5	27.03	145.67	230.33	45.33
